# MuST: multiple-modality structure transformation for single-cell spatial transcriptomics

**DOI:** 10.1093/bib/bbaf405

**Published:** 2025-08-28

**Authors:** Zelin Zang, Liangyu Li, Yongjie Xu, Chenrui Duan, Yue Shen, Yi Sun, Zhen Lei, Stan Z Li

**Affiliations:** Westlake Institute for Advanced Studies, Westlake University, HangZhou, 310000, China; Centre for Artificial Intelligence and Robotics (CAIR), HKISI-CAS, 310000; Westlake Institute for Advanced Studies, Westlake University, HangZhou, 310000, China; Westlake Institute for Advanced Studies, Westlake University, HangZhou, 310000, China; Westlake Institute for Advanced Studies, Westlake University, HangZhou, 310000, China; Ant Group, Hangzhou, 310000, China; Westlake Institute for Advanced Studies, Westlake University, HangZhou, 310000, China; Centre for Artificial Intelligence and Robotics (CAIR), HKISI-CAS, 310000; State Key Laboratory of Multimodal Artificial Intelligence Systems (MAIS), Institute of Automation, University of Chinese Academy of Sciences (CASIA), 17W, Science Park West Avenue, Hong Kong Science Park, Pak Shek Kok, New Territories, 999077, Hong Kong; School of Artificial Intelligence, University of Chinese Academy of Sciences (UCAS), Beijing 100049, China; School of Computer Science and Engineering, the Faculty of Innovation Engineering, Macau University of Science and Technology, Macau 999078, China; Westlake Institute for Advanced Studies, Westlake University, HangZhou, 310000, China

**Keywords:** modality bias, spatial transcriptomics (ST), multi-modality integration, topology discovery, biomarker identification

## Abstract

Spatial transcriptomics (ST) technologies have revolutionized the study of gene expression patterns in tissues by providing multimodal data, including transcriptomic (Tra.), spatial, and morphological modalities, thereby offering new opportunities to understand tissue biology beyond traditional Tra. However, we identify the modality bias phenomenon in ST data species, i.e. the inconsistent contribution of different modalities to the labels leads to a tendency for the analysis methods to retain the information of the dominant modality. How to mitigate the adverse effects of modality bias to satisfy various downstream tasks remains a fundamental challenge. This paper introduces Multiple-modality Structure Transformation, named MuST, a novel methodology to tackle the challenge. MuST integrates the multi-modality information contained in the ST data effectively into a uniform latent space to provide a foundation for all the downstream tasks. It learns intrinsic local structures by topology discovery strategy and topology fusion loss function to solve the inconsistencies among different modalities. Thus, these topology-based and deep learning techniques provide a solid foundation for a variety of analytical tasks while coordinating different modalities. The effectiveness of MuST is assessed by performance metrics and biological significance. The results show that it outperforms existing state-of-the-art methods with clear advantages in the precision of identifying and preserving structures of tissues and biomarkers. MuST offers a versatile toolkit for the intricate analysis of complex biological systems.

## Introduction

While single-cell RNA sequencing captures gene expression profiles of organisms at the single-cell resolution [1–4], more recent advances in spatial transcriptomics (ST) technology provide additional multiple modality information about the spatial (Spa.) and morphology (Mor.) patterns of tissues [5, 6]. The breakthrough technologies for ST, such as 10$\times $ Visium [7], Slideseq [8], SlideseqV2 [9], and Stereo-seq [10] that measure gene expression information and graphical information in captured locations (referred to as “spot”) at a resolution of several cells or even sub-cells levels. These advancements offer great potential for understanding, interpreting, and visualizing the intricate micro-structures and underlying biological processes within tissues [11–14]. The ST technology has been used to tackle a variety of downstream analysis tasks including (i) Spa. clustering [15, 16], (ii) spot visualization [17, 18], (iii) Spa. deconvolution [19, 20], (iv) marker gene analysis [21], and (v) Spa. trajectory inference [22].

We find that ST multiple modality data suffer from the modality bias phenomenon (details in the “Results” section). The modality bias phenomenon in ST data is similar to the concepts of those in the image and language fields [23, 24], which refers to the inconsistent contribution of different modality data to the label. For example, gene expression information is considered much more important than Mor. information [15, 25, 26]. However, for some complex tissue structures, the Mor. data can significantly improve the sharpness of the embedding and the precision of the biological analysis. Similarly, for some ST techniques that do not incorporate Mor. data, a better balance between Spa. location and gene expression also improves the performance of the method. Unresolved modality bias leads to substantial information loss and hampers the comprehensive utilization of multi-modal ST data in integrative downstream analyses. Addressing this bias is therefore crucial for improving analytical robustness and biological interpretability.

Existing ST methods fall into two main categories: clustering-based and (dis)similarity-based techniques. Clustering-based techniques, such as those based on contrastive learning [15, 27, 28] and deep embedding clustering [16, 27, 29–32], focus on preserving global information. Regardless of the severity of the modality bias, such methods discard local information, which leads to the failure of such methods in downstream tasks that require accurate local information (such as spot visualization and Spa. deconvolution). The (dis)similarity-based techniques, including kernel learning [25, 33–35] and distance fitting [36] methods, train models on similarities or dissimilarities. These methods use fixed weighting coefficients between different modalities to estimate the contribution in the data fusion process and neglect the important information in weaker modalities. We also include BINRES [32], a Bayesian method that adaptively integrates multi-modal Spa. data for clustering, though it does not provide a general latent representation for downstream tasks as Multiple-modality Structure Transformation (MuST) does. In addition, a fixed similarity also leads to local conflicts between the spots.

**Figure 1:**
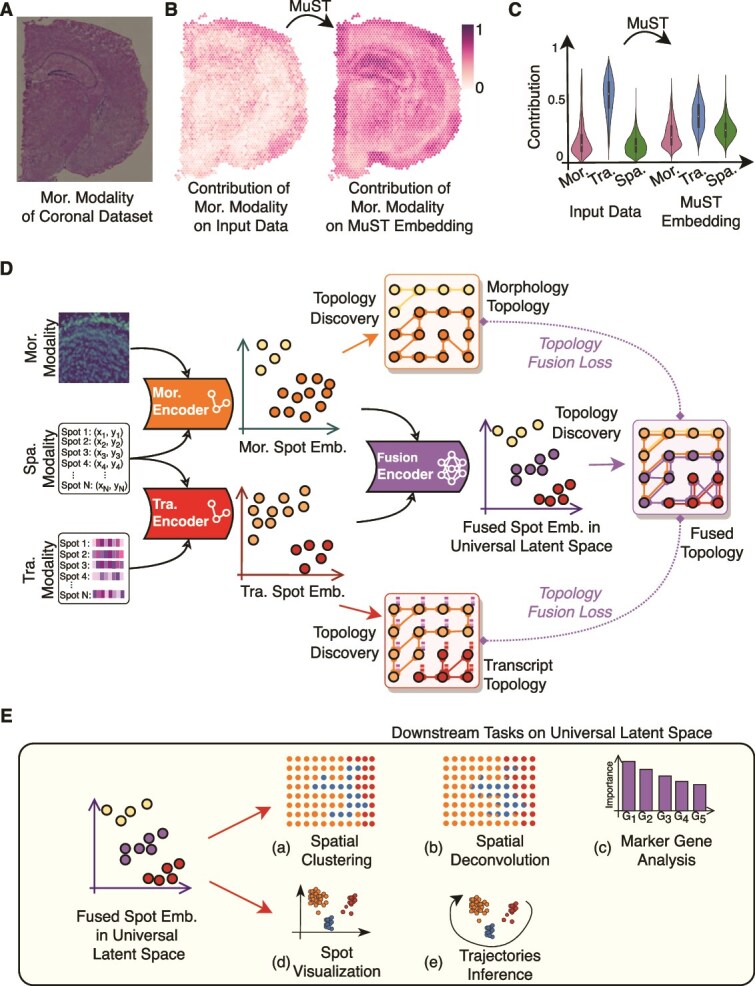
Illustration of MuST. (A) Mor. modality data of the coronal dataset. (B) Illustration of the modality bias phenomenon. In the case of Mor. modality, there is a significant inconsistency in the contribution of Mor. data to the final result (detected by Shapley value [43]). This inconsistency causes many methods to discard weaker modalities such as Mor. and Spa. resulting in a loss of information. MuST improves the retention of weaker modalities by mapping the Mor. modality to the Tra. modality. The results of the interpretable analyses show that the phenomenon of modality bias is mitigated by the Mor. Encoder processing. (C) The statistical evidence of the modality bias phenomenon is mitigated. (D) The ST data and its topologies of Tra. modalities, Mor. modalities, and Spa. modalities are fused into a universal latent feature space (universal latent space) in which the information loss problems caused by the modality bias phenomenon are mitigated and the topologies of multimodal data are well represented. Specifically, the unit of ST data is called a “spot,” and each spot contains Tra. data, Spa. data, and Mor. data. (E) Multiple downstream tasks. MuST incorporates information from multiple modalities and better supports multiple downstream tasks. These include (a) Spa. clustering, (b) spot visualization, (c) Spa. deconvolution, (d) marker gene analysis, and (e) Spa. trajectory inference. The mutual support of multiple downstream tasks allows for a more comprehensive understanding of ST data.

We introduced the MuST combining augmentation-based manifold learning [37, 38] with graph neural networks (GNNs) [39], which creates a universal latent space that integrates multiple input modalities and facilitating in-depth analysis of ST data across various downstream tasks. To mitigate the adverse effects of the modality bias phenomenon, MuST do not directly fuse the information of modalities, instead of measuring the modality’s importance in Mor. and transcriptomic (Tra.) spot embedding space (Fig. 1D). Specifically, we proposed a topology discovery strategy to accurate estimation global and local information of multiple modality data by extracting the topology from Mor./Tra. spot embedding space. In addition, a topology fusion loss is proposed to minimize the topological differences between all modalities and the latent space with an adaptive motility’s importance estimated from the continuously updated Mor./Tra.’s spot embedding. The effectiveness of MuST is evaluated comprehensively in terms of scientific indicators and biological significance by comparison with state-of-the-art methods on ST data of human and mouse tissues generated by different platforms (e.g. 10$\times $ Visium, Slide-seqV2, and Stereo-seq). On the scientific metrics, MuST has a clear advantage on the geometric structure preservation metrics and demonstrates superior or similar results on a range of clustering metrics. In terms of biological significance, MuST demonstrates more accurate spot clustering, spot visualization, and Spa. trajectory inference. In addition, the gene markers found by MuST are also validated by the published papers [40–42].

## Results

### Overview of MuST

**Figure 2:**
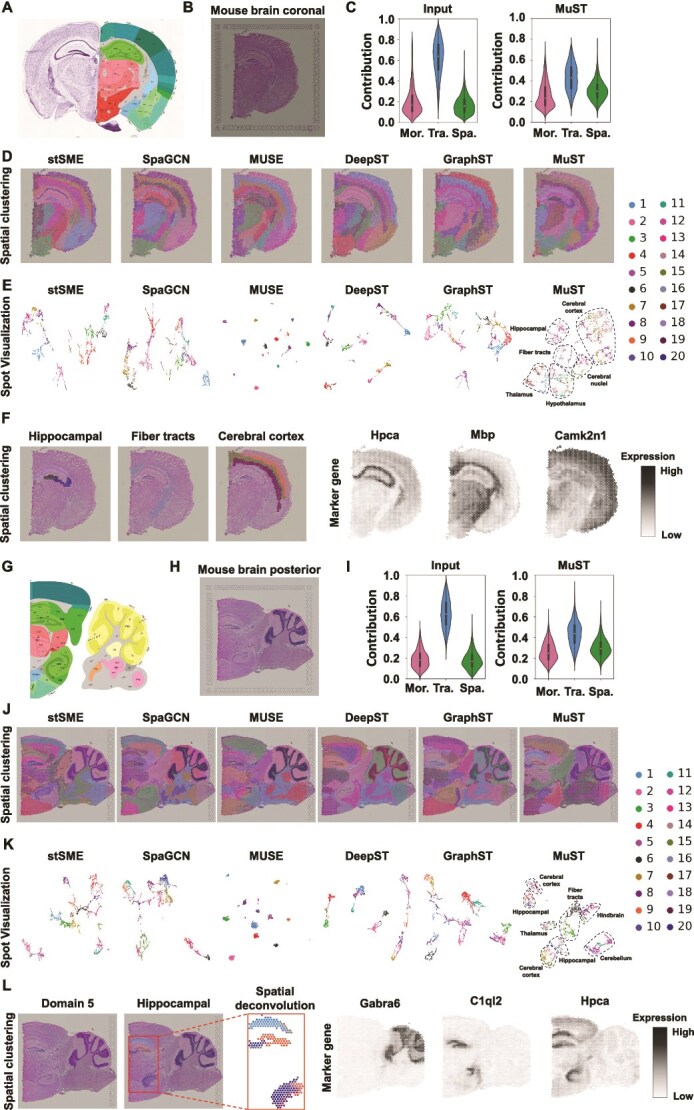
MuST explores more biologically complex tissues in adult mouse brain section profiled by 10$\times $ Visium (10$\times $ Genomics). (A) The annotation of hippocampus structures from the Allen Reference Atlas of an adult mouse brain. (B) H&E image of mouse brain coronal section. (C) Contribution of the Mor. modality, transcriptome (Tra.) modality, and Spa. modality input data or MuST embedding to data labeling. (D) Clustering results by Spa. methods, stSME, SpaGCN, MUSE, DeepST, GraphST, and MuST. (E) Spot visualization generated by the Spa. methods. (F) Single cluster visualization of Spa. domains identified by MuST and the corresponding marker gene expressions. (G) Allen Brain Institute reference atlas diagram of the mouse sagittal. (H) H&E image of mouse sagittal posterior brain section. (I) Contribution of the Mor. modality, Tra. modality, and Spa. modality input data or MuST embedding to data labeling. (J) Clustering results by Spa. methods, stSME, SpaGCN, MUSE, DeepST, GraphST, and MuST. (K) Spot visualization generated by the Spa. methods. (L) Single cluster visualization of Spa. domains identified by MuST and the corresponding marker gene expressions and cluster-related Spa. deconvolution.

**Figure 3:**
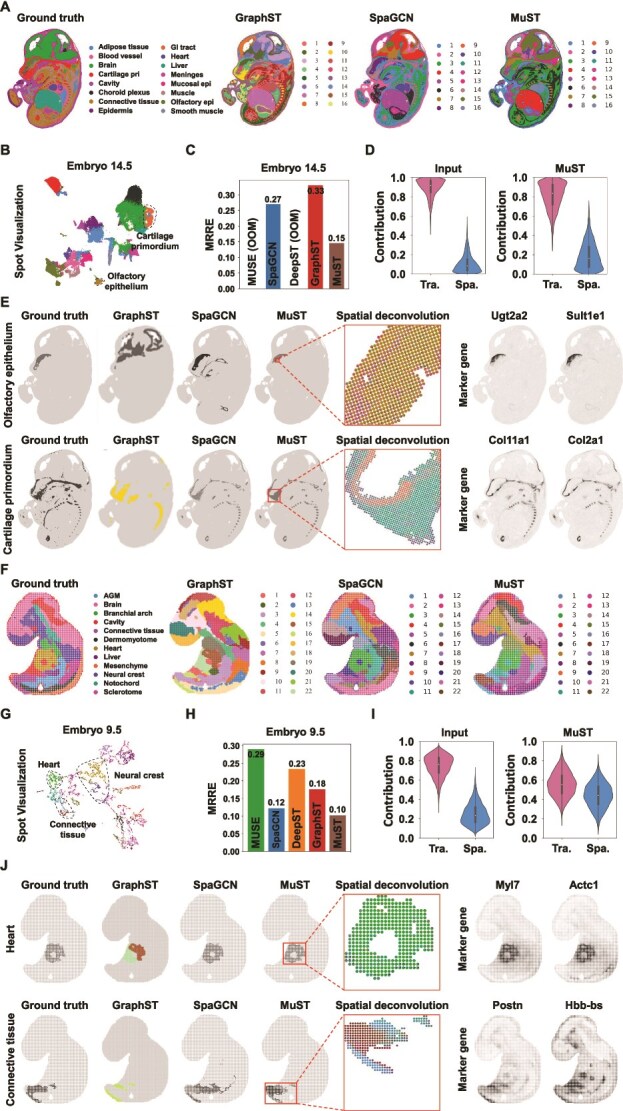
MuST enables accurate identification of different organs in the Stereo-seq mouse embryo (MOSTA). (A) Tissue domain annotations obtained from the original Stereo-seq study and clustering results on the E14.5 mouse embryo data. (B/G) Spot visualizations generated by MuST representations. (C/H) MRRE scores generated by baseline representations. Among them, methods MUSE and DeepST have an out-of-memory problem. (D/I) Contribution of the transcriptome (Tra.) modality and Spa. modality input data or MuST embedding to data labeling. (E/J) Single cluster visualization of selected Spa. domains identified by the original Stereo-seq study. (F) Tissue domain annotations obtained from the original Stereo-seq study and clustering results on the E9.5 mouse embryo data.

We observe that ST data suffer from the modality bias phenomenon. As shown in Fig. 1A–C, the contributions of the Mor., Tra., and Spa. modalities to the data labels are significantly inconsistent. We use the interpretable method (Shapley Value [43, 44]) to show the differences in the contribution of data from different modalities to the ground truth (Section “Detecting modality’s contributions to labels using Shapley Value”). As shown in Fig. 1B, in the coronal mouse brain dataset (Coronal), the contribution of the Mor. modality is not consistent, and in most regions the contribution of Mor. modality is negligible. However, in some specific tissue structures (e.g. hippocampus), the Mor. modality provides a stronger contribution. Furthermore, the violin plots in Fig. 1C show the statistical evidence of the modality bias phenomenon and its mitigation by MuST. The modality bias phenomenon causes some methods to ignore weaker modalities such as Mor. and Spa., resulting in a loss of information. Additional evidence from Figs 2 and 3 indicates that modality bias in modality contributions is a common issue in ST data.

We propose MuST, a robust augmentation-based manifold learning ST method for several downstream tasks by fusing multiple modalities. MuST mitigates the modality bias phenomenon by introducing topology knowledge and a topology fusion loss function (evidence in Fig. 1B). MuST plays a crucial role in balancing the contributions of different modalities, thereby reducing information loss. The framework of MuST is shown in Fig. 1D. Specifically, MuST first fuses the Spa. information into the Mor./Tra. spot embedding space by the Mor./Tra. encoder. Then we fuse both Mor./Tra. spot embedding spaces into the universal latent space (fused spot embedding space) with the fusion encoder. We use a topology discovery strategy to accurately estimate global and local information for different modalities to discover the local connection in Mor./Tra. embedding spaces based on $k$-nearest neighbor ($k$NN). The superposition of local connections is then used to describe the global relationships of the data, i.e. a non-neighboring point can be described by passing through several levels of neighborhood relationships. To make the data distribution in the representation space more realistic, we dynamically generate new samples using neighborhood-based interpolation and use the generated spots in the training process. Finally, we design topology fusion loss functions to fuse local and global information from different modalities using augmented data (Section “Framework of MuST”). In our framework, we consider Mor., Tra., and Spa. coordinates (Spa.) as three modalities. While Spa. is not a direct measurement like Mor. or Tra., it plays a fundamental role in defining Spa. adjacency and guiding local information propagation in downstream analyses. Therefore, Spa. is treated as a functional modality in MuST.

The universal latent space learned by MuST combines local and global information from different modalities to perform downstream tasks (Fig. 1E). Specifically, a Spa. clustering task using clustering methods (e.g. mclust [45]), a spot visualization task using visualization methods (e.g. an MLP network based on topology loss [46] and UMAP [47]), marker gene analysis task using sensitivity analysis [48], Spa. trajectory inference task using PAGA [22] method, and Spa. deconvolution task using Lasso [49] method on the clustering results. The downstream tasks corroborate each other and provide a better understanding of the ST data. We compare MuST with existing methods, such as GraphST [15], STAGATE [25], DeepST [35], SpaGCN [31], MUSE [16], and stSME [33], evaluating performance based on metrics including mean relative rank error (MRRE), adjusted Rand index (ARI), and biological significance analysis.

### MuST mitigates modality bias and recognizes complex tissues on mouse brain data

We initially assess MuST’s performance by its effectiveness in mitigating modality bias phenomenon and biological significance in mouse brain data. This evaluation confirms MuST’s proficiency in accurately identifying complex tissue structures. The datasets comprise three distinct modalities: Tra., Mor., and Spa., from two specific sections of the mouse brain—coronal mouse brain section and a mouse sagittal posterior brain section (Fig. 2B and H), acquired using the 10$\times $ Visium platform. We compare the Spa. domains identified by MuST with the corresponding anatomical reference annotations from the Allen Mouse Brain Atlas [50] (Fig. 2A and G), the goal is to verify our findings and assess the interpretative precision of MuST. Moreover, to ensure a higher resolution in our Spa. clustering task, we set the number of clusters to $20$ for both datasets.

For the coronal mouse brain section, MuST better examine the hippocampal region, fiber tract region, cerebral cortex region, and V3 structure. The Spa. domains identified by baseline methods roughly partition the tissue structures into groups containing different cell types while unable to identify small Spa. domains (Fig. 2D). Specifically, all baseline methods fail to identify the V3 structure and “cordlike” structure (Ammon’s horn). stSME, SpaGCN, and DeepST fail to identify the “arrow-like” structure (dentate gyrus within the hippocampus). stSME, SpaGCN, MUSE, and DeepST fail to identify some small fiber tracts. In contrast, MuST brings noticeable enhancements in Spa. domain identification (Fig. 2D). As follows, (i) in the hippocampal region, MuST identifies the CA1 field (domain 18) and CA3 field (domain 20) of the Ammon’s horn and the dentate gyrus structure (domain 6). The identified hippocampal region has high concordance with the selected marker genes Hpca (Fig. 2F; Supplementary Table S9 and Fig. S10). The quantitative comparison of Fig. 2D and J with MRRE are shown in Supplementary Fig. S10. These advantages may be attributed to the significant contribution of Tra. modality after MuST to Ammon’s horn and the dentate gyrus structure (Supplementary Fig. S2C). (ii) MuST better depicts the fiber tracts and groups the fiber tracts with texture structure in the histological image into one cluster (domain 16), which has high concordance with the selected marker genes Mbp (Fig. 2F). (iii) In the cerebral cortex region, MuST identifies more cortical layers, including external (Layers 2/3), internal (Layers 4 and 5), and plexiform (Layer 6) layers, and the thickness of the cerebral cortex is highly consistent with the anatomical reference annotations. This advantage is due to the combined contribution of Tra. modality (e.g. Layers 4 and 6) and Mor. modality (e.g. Layers 2/3 and 5) after MuST to the cerebral cortex (Supplementary Fig. S2C), thus validating the ability and significance of MuST to mitigate modality bias phenomenon (Fig. 2C). The identified cerebral cortex region concurs highly with the selected marker gene Camk2n1 (Fig. 2F). (iv) MuST accurately identifies the V3 structure (domain 4), which is not possible with baseline methods. The Fabp7 gene expression is well alignment with the identified V3 structure (Supplementary Fig. S10). In the visualization results (Fig. 2E), MuST also separates these tissue structures marked with a dashed circle. In the Spa. deconvolution task, the cluster compositions inferred by MuST also accurately depict the regions identified by MuST (Supplementary Fig. S6C and E).

For the mouse sagittal posterior brain section, MuST better examines the hippocampal region and coronal structure. The baseline methods cannot also identify small Spa. domains (Fig. 2J). Specifically, they fail to identify the “cordlike” structure, “arrow-like” structure, and the thin layer around the coronal structure. In contrast, MuST better alleviates the problems existing in these methods (Fig. 2J). (i) In the hippocampal region, MuST identifies the CA1 field (domain 1) and CA3 field (domain 20) of the Ammon’s horn, which has high concordance with marker gene Hpca (Fig. 2L; Supplementary Table S10 and Fig. S11). MuST can group the dentate gyrus structure (domain 9) into one cluster with high concordance with the selected marker gene C1ql2 (Fig. 2L). The cluster compositions inferred by MuST also accurately depict the identified hippocampal region (Fig. 2L and Supplementary Fig. S6D and F). These advantages may be attributed to the significant contribution of Tra. modality after MuST to “cordlike” structure and “arrow-like” structure (Supplementary Fig. S2C). (ii) MuST can depict the coronal structure and thin layer around it (domains 5, 11, and 12), and MuST is the only method that can clearly distinguish these three domains, which correspond to the texture structure of histological image. This advantage is due to the combined contribution of Tra. modality (e.g. thin layer around the coronal structure) and Mor. modality (e.g. coronal structure) after MuST to this region (Supplementary Fig. S2C), thus validating the ability and significance of MuST to mitigate modality bias phenomenon (Fig. 2I). The Gabra6 expression aligns well with the identified Spa. domain (Fig. 2L). In the visualization results, MuST significantly improves the separation of tissue structures (e.g. cerebellum, thalamus, cerebral cortex, hindbrain, and fiber tracts) (Fig. 2K) mentioned above, this verifies that the universal latent space, which can better fuse information from multiple modalities, is beneficial for various downstream tasks.

### MuST distinctly recognizes fine-grained tissue structures on mouse embryo data

Building on our preliminary evaluation of MuST in mouse brain data, which involves assessing its capability to mitigate modality bias across three modalities and its biological significance. We now broaden our research scope to encompass Stereo-seq [10] datasets from mouse embryos at developmental stages E14.5 (Fig. 3A) and E9.5 (Fig. 3F). This phase of evaluation specifically concentrates on mitigating modality bias, particularly in Tra. modality and Spa. modality, within the context of large-scale data. The tissue domain annotations for these datasets are obtained from the original study [10].

For the E14.5 mouse embryo, MuST better recovers the olfactory epithelium and cartilage primordium region. We set the number of clusters to $16$ to match the original annotation for the Spa. clustering task (Fig. 3A). MuST, GraphST, and SpaGCN capture much of the fine-grained structure in the embryo and accurately identify major areas, such as the heart, liver, muscle, epidermis, and meninges regions (Fig. 3A). Furthermore, MuST captures more of the fine-grained structures (Supplementary Fig. S4C), such as the olfactory epithelium and cartilage primordium regions (Fig. 3E). MUSE and DeepST could not output results because too many data points caused memory problems. As follows, (i) MuST better demarcates olfactory epithelium as a separate region [15], consistent with the original annotation, whereas GraphST clusters it as part of the brain region and SpaGCN assigns part of the mucosal epithelium region to it. In the visualization space (Fig. 3B and Supplementary Fig. S7A), MuST also separates the olfactory epithelium region labeled by a dashed circle [46]. The identified olfactory epithelium region has high concordance with the selected marker genes Ugt2a2 and Sult1e1 (Fig. 3E and Supplementary Table S6). The cluster compositions inferred by MuST accurately depict the identified olfactory epithelium region (Fig. 3E). These advantages may be attributed to the significant contribution of Tra. modality after MuST to olfactory epithelium region (Supplementary Fig. S3B). (ii) MuST better demarcates the cartilage primordium region around the brain, which has high concordance with the selected marker genes Col11a1 and Col2a1 (Fig. 3E). MuST also separates the cartilage primordium region labeled by a dashed circle (Fig. 3B). The cluster compositions inferred by MuST accurately depict the identified cartilage primordium region (Fig. 3E). In addition, MuST obtains the best MRRE score $0.15$ compared with GraphST ($0.33$) and SpaGCN ($0.27$) (Fig. 3C).

For E9.5 mouse embryo, MuST better recovers the heart, neural crest, connective tissue, and notochord regions. Although the original annotation has $12$ reference clusters (Fig. 3F), the number of clusters in our testing is set to $22$ for the Spa. clustering task to acquire a higher resolution of tissue segmentation [15]. Some clusters of GraphST and SpaGCN match the annotated regions (Fig. 3F), such as liver region, but the contours of mesenchyme, sclerotome, and AGM regions are not accurate. In contrast, MuST further captures much of the fine-grained and contours structures, including heart, neural crest, notochord, connective tissue, brain, and cavity regions (Fig. 3F and Supplementary Fig. S5C), and are highly concordant with the original annotation. As follows, (i) MuST demarcates the heart region as a separate cluster, consistent with the original annotation, whereas GraphST divides it into two clusters. In the visualization results, MuST also separates the heart region labeled by a dashed circle (Fig. 3G and Supplementary Fig. S7B). The identified heart region has high concordance with the selected marker genes Myl7, Actc1, and Acta1 (Fig. 3J and Supplementary Table S7). The cluster compositions inferred by MuST accurately depict the identified heart region (Fig. 3J). These advantages may be attributed to the significant contribution of Tra. modality after MuST to heart region (Supplementary Fig. S3B). (ii) MuST better describes the contours of the notochord region and more completely demarcates the connective tissue region, whereas SpaGCN assigns part of the AGM region to connective tissue region. The identified connective tissue region concurs highly with the selected marker genes Postn and Hbb-bs (Fig. 3J). This advantage is due to the combined contribution of Tra. modality and Loc. modality after MuST to connective tissue region (Fig. 3I and Supplementary Fig. S3B). (iii) MuST better demarcates the neural crest region, whereas GraphST assigns part of the mesenchyme and brain regions to this region. In addition, MuST obtains best MRRE score $0.10$, followed by SpaGCN ($0.12$) and GraphST ($0.18$) (Fig. 3H and Supplementary Table S2). The evaluation of universal latent space representations helps us to explain the above analysis and verifies the effectiveness of modality fusing ability in MuST.

### MuST distinctly recognizes anatomical regions on mouse hippocampus data

**Figure 4:**
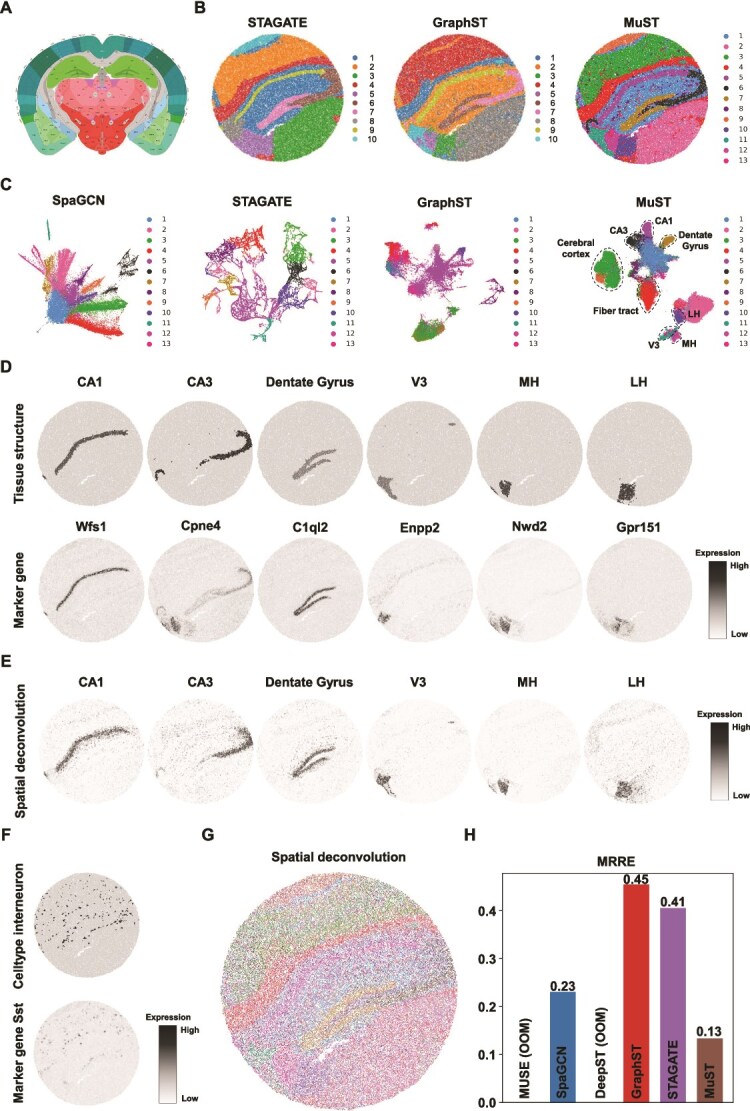
MuST discerns relevant anatomical regions more accurately in the SlideseqV2 mouse hippocampus data (SCP354). (A) Allen Brain Institute reference atlas diagram of the mouse cortex. (B) Clustering results by Spa. methods, STAGATE (original paper), GraphST (original paper), and MuST on the mouse hippocampus data. (C) Spot visualizations generated by SpaGCN, STAGATE, GraphST, and MuST representations. (D) Single cluster visualization of the tissue structures identified by MuST and the corresponding marker gene expressions. (E) Cluster-related Spa. deconvolution analysis of the tissue structures identified by MuST. (F) Visualization of the celltype identified by MuST and the corresponding marker gene expressions. (G) Cluster-related Spa. deconvolution analysis. (H) MRRE scores generated by SpaGCN, GraphST, STAGATE, and MuST representations.

Building on the insights gained from the analysis of the 10$\times $ Visium and Stereo-seq platform, we extend our investigation to more advanced platform SlideseqV2 [9]. This progression allows us to explore the consistency of ST patterns across different technological iterations and the robustness of MuST. For this comparison, we employ the annotated Allen Brain Atlas as the ground truth [50] (Fig. 4A).

MuST produces more spatially consistent clustering and captures major anatomical regions, such as the dentate gyrus and the pyramidal layers within Ammon’s horn (CA1 and CA3 regions), and MuST better discerns the cavity tissue structure and the region covered by interneuron cells. We set the number of clusters in our testing to $13$ to acquire a higher resolution of tissue segmentation (Fig. 4B). Specifically, (i) MuST and GraphST are better than STAGATE in delineating the CA3 and dentate gyrus regions with sharper boundaries. MuST and STAGATE demarcate more neocortical layers in the cerebral cortex, and GraphST groups them into one region. (ii) MuST and GraphST can differentiate between the third ventricle (V3), medial habenula (MH), and lateral habenula (LH), which have high concordance with their marker genes Enpp2, Nwd2, and Gpr151, respectively (Fig. 4D; Supplementary Table S8 and Fig. S9). The cluster compositions inferred by MuST accurately depict the identified V3, MH, and LH regions (Fig. 4G and E). However, STAGATE merges the MH and LH into one region. For the V3 structure, the Enpp2 expression does not align well with the detected V3 region of MuST (Fig. 4D), but the latter better resembles the V3 region in the annotated brain reference (Fig. 4A). For comparison, the V3 regions of STAGATE are closer to the shape of the Enpp2 expression region but do not match the anatomical shape well. In the visualization results, MuST also separates the V3, MH, and LH regions (Fig. 4C). (iii) MuST can discern the cavity tissue structure and interneuron cell regions. The cavity region has higher concordance with the anatomical annotation (Fig. 4A), and the interneuron cell region concurs more with the selected marker gene Sst (Fig. 4F). Then, we use the MRRE score to explain the above analysis quantitatively (Fig. 4H and Supplementary Table S2). MuST obtains the best MRRE score $0.13$, while GraphST ($0.45$) and STAGATE ($0.41$) get much worse performance.

### MuST recognizes laminar structure on mouse olfactory bulb data

Building on the discussion of the block structure analysis performed on three different platforms, we now turn our attention to evaluating the capabilities in analyzing hierarchical/laminar structures. Because a clear delineation of the laminar structure can only be achieved by reasonably fusing the information provided by different modalities.

**Figure 5:**
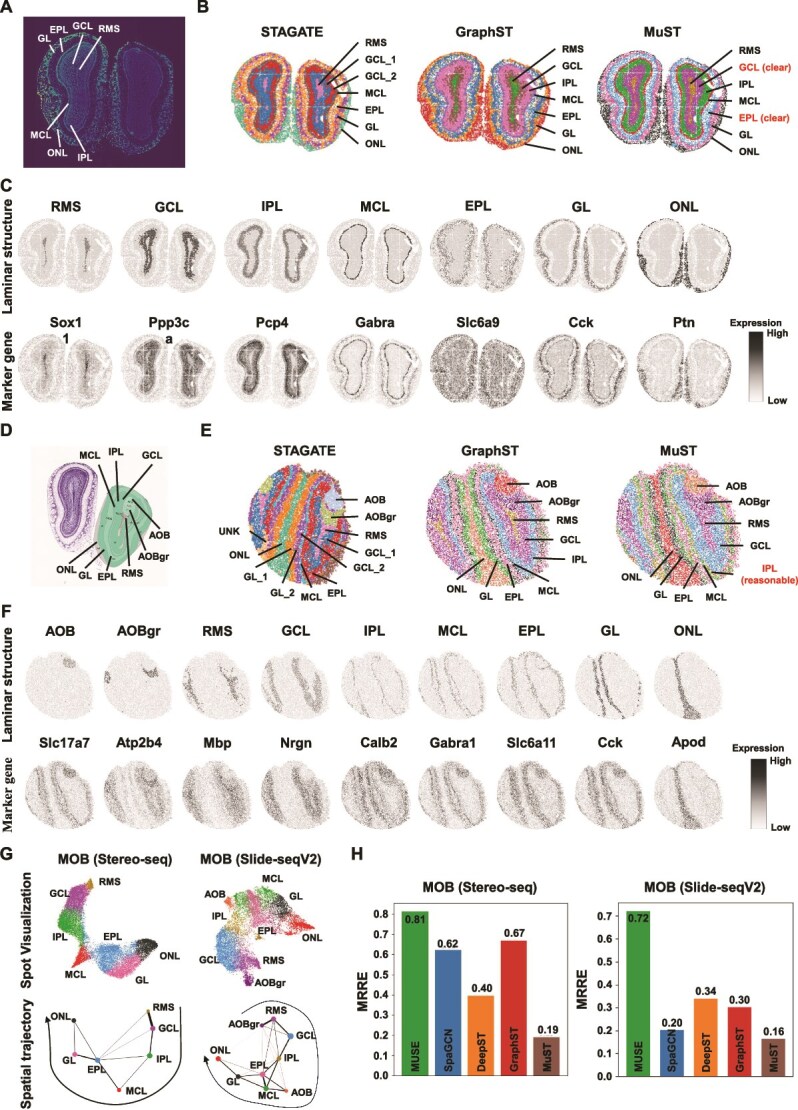
MuST identifies the laminar organization in the mouse olfactory bulb tissue sections profiled by Stereo-seq (SEDR) and Slide-seqV2 (SCP815), respectively. (A) Laminar organization of mouse olfactory bulb annotated in the DAPI-stained image generated by Stereo-seq. (B) Clustering results by Spa. methods, STAGATE (original paper), GraphST (original paper) and MuST on the Stereo-seq mouse olfactory bulb tissue section. (C) Single cluster visualization of the Spa. domains identified by MuST and the corresponding marker gene expressions. (D) Laminar organization of mouse olfactory bulb annotated by the Allen Reference Atlas. (E) Clustering results by Spa. methods, STAGATE (original paper), GraphST, and MuST on Slide-seqv2 mouse olfactory bulb tissue section. (F) Single cluster visualization of Spa. domains identified by MuST and the corresponding marker gene expressions. (G) Spot visualizations and PAGA graphs generated by the representations of MuST. (H) MRRE scores generated by MUSE, SpaGCN, DeepST, GraphST, and MuST representations.

We use two mouse olfactory bulb tissue datasets acquired with high-resolution platforms Stereo-seq [10] and Slide-seqV2 [9], to rigorously test MuST’s proficiency in delineating intricate laminar architectures. For the former, we compare the Spa. domains identified by MuST with the mouse olfactory bulb’s laminar structure annotated by the DAPI-stained image [25, 51], identifying the olfactory nerve layer (ONL), glomerular layer (Spa.), external plexiform layer (EPL), mitral cell layer (MCL), internal plexiform layer (IPL), granule cell layer (GCL), and rostral migratory stream (RMS) (Fig. 5A). For the latter, we compare the Spa. domains identified by MuST with the laminar organization of mouse olfactory bulb annotated by the Allen Reference Atlas [50], identifying the accessory olfactory bulb (AOB), granular layer of the accessory olfactory bulb (AOBgr), RMS, GCL, IPL, MCL, EPL, GL, and ONL (Fig. 5D).

For mouse olfactory bulb tissue section acquired with Stereo-seq, MuST more clearly recognizes the external and internal structure of the organ. We set the number of clusters to $7$ to match the known laminar structure for the Spa. clustering task. STAGATE, GraphST, and MuST can separate the outer layers of the organ, namely the ONL, GL, and EPL, but STAGATE seriously confuses GL and EPL structures (Fig. 5B). MuST and GraphST can demarcate the GCL and RMS structures for the inner structure. However, GraphST’s clusters are noisy, and the boundaries between different structures are not as straightforward as MuST. We use the respective selected marker genes of each anatomical region to validate MuST’s clusters (Fig. 5C), and find the excellent correspondence between MuST’s clusters and the known marker genes (Supplementary Table S11 and Fig. S12), such as Gabra1 [40], Cck, and Ptn. For some marker genes (e.g. Ppp3ca and Pcp4), their high expression levels overlap with neighboring regions. This is expected as cell types are often shared among the different inner structures of organs, and markers are likewise shared among similar cell types. MuST delineates the Spa. trajectory among the mouse olfactory bulb (from RMS to GCL to ONL) with clear boundaries between different structures in the visualization results as well as the PAGA graphs [22] (Fig. 5G), but other methods do not have this capability (Supplementary Fig. S7C). In addition, MuST obtains the best MRRE score 0.19 (Fig. 5H and Supplementary Table S2), while MUSE (0.81) and GraphST (0.67) get much worse performance.

For mouse olfactory bulb tissue section acquired with Slide-seqV2, MuST better recognizes the laminar structure using fewer clusters. We set the number of clusters to $9$ to match the known laminar structure for the Spa. clustering task. The Spa. domains identified by MuST are consistent with the mouse olfactory bulb annotation from the Allen Reference Atlas. At the same time, baseline methods need more clusters to identify these structures (Fig. 5E). These Spa. domains uncovered by MuST are clearly supported by known and selected gene markers (Fig. 5F; Supplementary Table S12 and Fig. S13). The granular cell marker Atp2b4 [52] shows strong expressions on the identified AOBgr domain. MuST identifies the GCL structure with the dominant expression of Nrgn [41]. Nrgn is a well-documented schizophrenia risk gene, implying that this domain is related to cognition function. The narrow MCL structure with the dominant expression of mitral cell marker Gabra1 [40] is also identified by MuST. MuST delineates the Spa. trajectory among the mouse olfactory bulb (from AOBgr to RMS to ONL) in the visualization results and the PAGA graphs (Fig. 5G), but other methods do not have this capability (Supplementary Fig. S7D). In addition, MuST obtains the best MRRE score of 0.16, while MUSE (0.72) and GraphST (0.48) get much worse performance (Fig. 5H and Supplementary Table S2). Collectively, these results illustrate the modalities fusing ability of MuST to identify tissue structures and reveal their organization from ST data of different Spa. resolutions.

### MuST exploratively recognizes layers on human dorsolateral prefrontal cortex data

Having established MuST’s efficacy in analyzing block structures in mouse data, we extend our investigation to human datasets to assess its broader applicability. This transition from model organisms to human data is crucial for validating MuST’s utility in more complex and clinically relevant settings.

**Figure 6:**
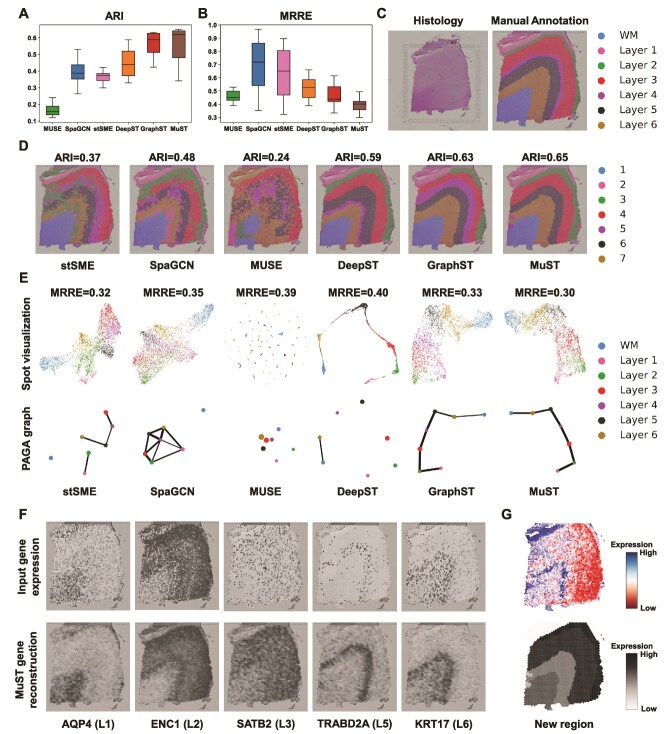
MuST improves the identification of layer structures in the human DLPFC tissue (spatialLIBD). (A) Boxplots of ARI scores of six Spa. methods applied to the 12 DLPFC slices. Boxplots denote medians and interquartile ranges (IQRs). The whiskers of a boxplot are the lowest datum still within 1.5 IQR of the lower quartile and the highest datum within 1.5 IQR of the upper quartile. (B) Boxplots of MRRE scores of six Spa. methods applied to the 12 DLPFC slices. (C) H&E image and manual annotation from the original study. (D) Clustering results by Spa. methods, stSME, SpaGCN, MUSE, DeepST, GraphST, and MuST on slice 151 673 of the DLPFC dataset. Manual annotations and clustering results of the other DLPFC slices are shown in Supplementary Fig. S8. (E) Spot visualizations and PAGA graphs generated by stSME, SpaGCN, MUSE, DeepST, GraphST, and MuST representations on slice 151 673. (F) Visualizations of the input gene expressions and MuST gene reconstructions of five layer-marker genes on slice 151 673. (G) Gene expressions analysis of the new regions.

We apply MuST to LIBD human dorsolateral prefrontal cortex (DLPFC) dataset [42], which contains ST profiles of $12$ DLPFC slices and each depicting the four or six layers of the human dorsolateral prefrontal cortex and white matter (WM). Across $12$ slices, for ARI score (Fig. 6A and Supplementary Table S3), MuST achieves the highest median score $0.62$, GraphST obtains the second median score $0.59$ after MuST, MUSE obtains the poorest median score $0.16$, and the remaining methods have median ARI scores of <0.44. For MRRE score (Fig. 6B), MuST achieves best median score $0.40$, GraphST obtains the second median score $0.44$, and the MRRE median scores of SpaGCN ($0.72$) and stSME ($0.65$) are worse. We illustrate the results with one slice 151 673 (Fig. 6D–G).

MuST achieves more competitive performance on Spa. clustering tasks than baseline methods. The visual comparisons clearly show that the MUSE performs the worst Spa. clustering, failing to identify every slice layer 151 673. stSME and SpaGCN slightly alleviate the problem existing in MUSE with the ability to recover WM, but with the remaining clusters mixed among six layers, including the WM has a broken boundary and being clustered together with a portion of Layer 6, and the boundaries between other clusters are also very chaotic with no clean separation. DeepST accomplishes better separation of each layer, but with incorrect layer thickness, fails to recover Layer 4, and the boundaries between WM and Layer 6 still need to be more apparent. GraphST and MuST produce layers that are significantly closer in shape to the manual annotation (Fig. 6C), but with some minor flaws. GraphST fails to recover Layer 2, and the thickness of Layers 1 and 4 are not accurate, while MuST successes to recover Layer 2 and better captures the position and thickness (Fig. 6D). MuST also fails to identify Layer 4, because Layer 4 is usually skinny and contains similar cell types to Layer 3, such as pyramidal neurons. Therefore, MuST discovers a new Spa. domain that differs significantly in gene expression from the others (Fig. 6G). For quantitative assessment, MuST achieves the highest ARI score of 0.65, GraphST obtains the second highest ARI score of 0.63, while the MUSE is the poorest method at an ARI score of 0.24. The results with all other slices are shown in Supplementary Fig. S8.

MuST effectively incorporates Spa. modality in the data, which allows it to reflect the distance between Spa. domains and capture Spa. trajectory in the visualization results [47]. For example, in slice 151 673, the different cortical layers appear well organized and follow a clear Spa. trajectory (from Layer 1 to Layer 6 to WM) in the visualization results (Fig. 6E). This result is consistent with the functional similarity between adjacent cortical layers and the chronological order [53]. By contrast, in the visualization results of MUSE embeddings, spots belonging to the same layers are split into multiple clusters. As for the other Spa. clustering methods, stSME mixes the spots of Layer 1 (green) and other cortex layers, and SpaGCN does not distinguish Layer 4 and other cortex layers clearly. We additionally validate the inferred Spa. trajectory utilizing PAGA (Fig. 6E). The PAGA graphs of MuST and GraphST representations show an almost linear development trajectory from Layer 1 to Layer 6 and the similarity between adjacent layers. In contrast, the PAGA result of both SpaGCN representations is mixed to different degrees, and the PAGA results of stSME, MUSE, and DeepST representations cannot recover Spa. trajectories. For quantitative assessment, MuST achieves the lowest MRRE score of 0.30, while the DeepST is the poorest method at an MRRE score of 0.40. In addition, MuST can denoize the gene expressions based on the universal latent space. We apply MuST to decrease noises in the DLPFC dataset to better show the Spa. pattern of genes across the layers of the cortex. We compare the expression profiles of five layer-marker genes of the input data to the denoized representations by MuST in the slice 151 673 in Fig. 6F. As anticipated, the MuST-denoized representations clearly show the laminar enrichment of these layer-specific marker genes. For example, after denoizing, the AQP4, ENC1, SATB2, TRABD2A, and KRT17 genes [42] show differential expressions in Layer 1, Layer 2, Layer 3, Layer 5, and Layer 6, respectively, which are consistent with previously reported results.

## Discussion

This paper introduces MuST, an augmentation-based manifold learning model that integrates gene expression, tissue Mor., and Spa. information into a latent space for a variety of downstream tasks, including Spa. clustering [15, 16], spot visualization [17, 18], Spa. deconvolution [19, 20], marker gene analysis [21], and Spa. trajectory inference [22], with improved precision.

MuST mitigates the detrimental effects of modality bias and therefore has a robust modality integration capability. The information in the weaker modalities (Mor. modality and Spa. modality) is better exploited to characterize and improve the performance of various downstream tasks. As an indication of the benefits of these techniques, MuST exploits morphological and textural differences to improve the identification of key tissue structures. For example, in the coronal mouse brain dataset and the sagittal mouse posterior brain dataset (Section “MuST mitigates modality bias and recognizes complex tissues on mouse brain data” and Fig. 2), MuST more accurately detects morphological variations, which sharpens the distinction of hippocampal region, cerebral cortex region, and coronal structure. However, GraphST [15] fails to identify the “string-like” and “arrow-like” structures due to its neglect of morphological information. The coarse-grained detection of hippocampal structure by other methods (e.g. stSME [33], SpaGCN [31], and DeepST [35]) may be due to the negative effect of the modality bias. Furthermore, in the human DLPFC dataset (Section “MuST exploratively recognizes layers on human dorsolateral prefrontal cortex data” and Fig. 6) [42], MuST more effectively recognizes the texture structure (cluster 2 in Fig. 6D) in histology images, while GraphST neglects this information. MuST is also better at recovering the fine-grained tissue structures in mouse embryo datasets due to the integration of Spa. and Tra. modalities (Section “MuST distinctly recognizes fine-grained tissue structures on mouse embryo data” and Fig. 3). It handles large data sets, although the stereo-seq technology does not provide data for the Mor. modality. However, the modality bias leads to inaccurate results from other methods (e.g. olfactory epithelium, cartilage primordium, heart, neural crest, notochord, connective tissue, brain, and cavity regions) compared with the ground truth.

Another advantage of MuST is its ability to perform synergistic analysis across multiple downstream tasks, potentially yielding consistent, confirmatory results. In Spa. clustering, MuST identifies distinct regions such as olfactory epithelium, cartilage primordium, heart, notochord, and connective tissue in embryo data (Section “MuST distinctly recognizes fine-grained tissue structures on mouse embryo data”), and hippocampal areas (e.g. CA1, CA3, dentate gyrus), fiber tracts, cerebral cortex and V3 structures in mouse brain data (Section “MuST mitigates modality bias and recognizes complex tissues on mouse brain data”). At the same time, some regions (e.g. olfactory epithelium, hippocampus, fiber tracts, cerebral cortex) are also delineated in the visualization subtask, revealing intricate details of tissue similarity. Building on this universal latent space, Spa. clustering, and spot visualization guide the interpretability and feature selection task to focus on specific genes associated with the identified differences. For example, the unity between the tagged regions and selected marker genes—Ugt2a2 and Sult1e1 in the olfactory epithelium region, Myl7 and Actc1 in the heart region (Section “MuST distinctly recognizes fine-grained tissue structures on mouse embryo data”), and C1ql2 and Hpca in the hippocampus region (Section “MuST mitigates modality bias and recognizes complex tissues on mouse brain data”)—is evident. The cluster compositions derived from MuST accurately characterize these regions. These downstream tasks show remarkable consistency in biological significance, outperforming other baseline methods.

Ultimately, MuST is versatile, processing ST data from a variety of platforms, such as 10$\times $ Visium [7], Slide-seqV2 [9], and Stereo-seq [10], and effectively managing the different Spa. resolutions inherent in these technologies. In the future, we plan to extend MuST to build a pre-trained base model to provide a foundation not only for the tasks considered in this work, but also for other related tasks, including solving batch effect problems through graph structure integration, annotating cell types, and interpreting developmental trajectories. Due to its broad applicability, flexibility and potential for extension, we anticipate that MuST will become an indispensable tool in ST research.

## Methods

### Data preprocessing

ST data include gene expression counts (Tra. modality), optional tissue Mor. images (Mor. modality), and Spa. coordinates (Spa. modality). First, as shown in Supplementary Fig. S1A, gene expression counts are log transformed and normalized for library size using SCANPY [54]. These counts are then standardized to zero mean and unit variance. The next step is to select the 3000 most variable genes [55] for the MuST model input. For tissue images, as shown in Supplementary Fig. S1B, we center and crop a $224 \times 224$ pixel area around the probe coordinates. These images are then processed by the ResNet50 architecture [56], pre-trained on the ImageNet collection, to distill a 2048-dimensional feature vector, excluding the final classification layer. To further refine the data, we apply PCA, capturing the top 50 principal components to represent spot Mor.

The ST data comprised two distinct components: Tra. modality $\boldsymbol{{X}}^{\text{tr}}$, encapsulating the refined gene expression profiles, and Mor. modality $\boldsymbol{{X}}^{\text{mo}}$. The Tra. modality $\boldsymbol{{X}}^{\text{tr}}=\{\boldsymbol{{x}}^{\text{tr}}_{n}\}_{n=1}^{N}$ consists of vectors $\boldsymbol{{x}}^{\text{tr}}_{n}$ in the space $\mathbb{R}^{{N_{t}}}$, with $N_{t}$ representing the count of selected genes. The Mor. modality $\boldsymbol{{X}}^{\text{mo}} = \{\boldsymbol{{x}}^{\text{mo}}_{n}\}_{n=1}^{N}$ encompasses the histological image features, with $\boldsymbol{{x}}^{\text{mo}}_{n}$ belonging to $\mathbb{R}^{N_{m}}$ and $N_{m}$ denoting the feature count. To facilitate the analysis of this multiple modalities dataset, we construct a heterogeneous graph $\boldsymbol{G}(\{\boldsymbol{E}^{p}\}, \{\boldsymbol{X}^{\text{tr}}, \boldsymbol{X}^{\text{mo}}\})$, where $\boldsymbol{E}^{p}$ are the sets of edges interlinking spots across Spa. dimensions. We use an $\epsilon $-radius approach [57], connecting points that are within a certain distance $\epsilon $.

### Detecting modality’s contributions to labels using Shapley Value

Modality bias phenomenon in ST data refers to the tendency of data to be overly biased toward one domain, often resulting from attempts to align features across different domains, leading to a loss of domain-specific discriminative information [23, 24]. We use Shapley Value [43, 44], an interpretable method, to determine how much the data for each modality contributes to the true label. In practice, we use the shap package (https://github.com/shap/shap) to explain a default linear SVC (https://scikit-learn.org/LinearSVC.html) model trained using multiple modalities data (or single modalities embedding of MuST) as input and real labels as output. We extract the resulting shap values and merge them by modality via max() to determine each modality’s contribution.

Specifically, whether the input data comprises three modalities (mouse brain Coronal, mouse brain Sagittal-Posterior, and DLPFC) or two modalities (mouse embryo, mouse hippocampus), we first train a linear SVC model to fit the mapping from multi-modal data to model outputs. We then use the shap package to interpret the model’s contribution from each input modality. Although some methods (including MuST) does not explicitly contain input Spa. information, Shapley values can still reveal the model’s attention to different modalities.

### Framework of MuST

To obtain a universal latent space that fuses information from different modalities to support various downstream applications, we design the MuST framework (Supplementary Fig. S1C). The MuST framework integrates, a data augmentation module $\mathcal{A}(\cdot )$, a multi-modality fusion network $\mathcal{F}(\cdot )$, and a decoding network $\mathcal{D}(\cdot )$. In addition, a topology fusion loss $\mathcal{L}_{\text{T}}(\cdot )$ and a reconstruction loss function $\mathcal{L}_{\text{R}}(\cdot )$ are used to effectively train these components.

#### Data augmentation module

The data augmentation module $\mathcal{A}(\cdot )$ produces novel augmented samples by exploiting the constructed heterogeneous graph. As shown in Supplementary Fig. S1D, augmented instances $\boldsymbol{x}^{+}$ are created to match the distribution of data across multiple modalities. The module generates new data by integrating the topological information with the input features.

For data $x \in \mathbb{R}^{N}$, with $N$ representing the feature count, the data augmentation module’s operation is defined as:


(1) \begin{align*}& \begin{aligned} & \boldsymbol{x}^{+} = \mathcal{A}_{\mathcal{U}}(\boldsymbol{x}) = (1-r_{u}) \cdot \boldsymbol{x} + r_{u} \cdot \boldsymbol{x}^{\text{hop}^{1}}, \\ & \boldsymbol{x}^{\text{hop}^{1}} \sim \text{Hop}^{1}(x, E), r_{u} \sim U(0, p_{U}) \end{aligned}\end{align*}

where $\boldsymbol{x}^{\text{hop}^{1}}$ is drawn from the 1-hop neighbors of $\boldsymbol{x}$ within the edge set $E$. The coefficient $r_{u}$ is the mixing parameter, while $p_{U}$ is the uniform distribution hyperparameter. The augmentation operations are performed in real time during network training, increasing the variability of the data and preserving the integrity of the feature semantics. During training, $\mathcal{H}_{ij}=0$ indicates the augmentation relationship between $\boldsymbol{x}_{j}$ and $\boldsymbol{x}_{i}$. For example, if $\mathcal{H}_{ij}=1$, then $\boldsymbol{x}_{j}$ is the augmented data of $\boldsymbol{x}_{i}$, id $\mathcal{H}_{ij}=0$, $\boldsymbol{x}_{j}$ is sampled from the same modality data set.


(2) \begin{align*}& \boldsymbol{x}_{i} \in \boldsymbol{X}^{\text{m}}, \boldsymbol{x}_{j} \sim \left\{ \begin{aligned} & \boldsymbol{x}_{j} \sim \boldsymbol{X}^{\text{m}} & \text{if} \ \ \ \mathcal{H}_{ij}=0 \\ & \boldsymbol{x}_{j} \sim \mathcal{A}(\boldsymbol{x}_{i}) & \text{if} \ \ \ \mathcal{H}_{ij}=1. \end{aligned} \right.\end{align*}

#### Multi-modality fusion network

The multi-modality fusion network includes Mor. Encoder, Tra. Encoder, and a Fusion Encoder. The Mor. Encoder, Tra. Encoders are based on GNN [39] and extract local structural features from the Mor. and Tra. modalities using Spa. coordinates. The Fusion Encoders are based on Multilayer Perceptron (MLP) [58] and fuse the information from the output of the Mor. Encoder and Tra. Encoder. The fusion process is described by the following equation:


(3) \begin{align*}& \begin{aligned} \boldsymbol{z} & = \text{MLP}_{\phi}(\boldsymbol{y}), \boldsymbol{y} = \text{Enc}_{\pi}(\text{cat}(\boldsymbol{y}^{\text{mo}}, \boldsymbol{y}^{\text{tr}})) \\ \boldsymbol{y}^{\text{mo}} & = \text{GNN}_{\theta}(\boldsymbol{x}^{\text{mo}}, E^{\text{p}}) \\ \boldsymbol{y}^{\text{tr}} & = \text{GNN}_{\omega}(\boldsymbol{x}^{\text{tr}}, E^{\text{p}}) \end{aligned}\end{align*}

where $\boldsymbol{x}^{\text{mo}}$ and $\boldsymbol{x}^{\text{tr}}$ represent the Mor. and Tra. data inputs, while $E^{\text{p}}$ denotes the Spa. coordinate graph structure. The resultant vector $\boldsymbol{z}$ resides in the Fused Spot embedding space (Fused Spot Emb). The vectors $\boldsymbol{y}^{\text{mo}}$ and $\boldsymbol{y}^{\text{tr}}$ occupy the Mor. and Tra. spot embedding spaces, respectively. The operation $\text{cat}$ denotes the concatenation of data. $\text{Enc}_{\pi }$ is the dedicated encoder for data fusion. The GNNs, $\text{GNN}_{\theta }$ and $\text{GNN}_{\omega }$, process Mor. and Tra. data, respectively.

The multi-modality fusion network is used to reconstruct the input data from the latent space :


(4) \begin{align*}& \begin{aligned} \boldsymbol{\hat{x}}^{\text{tr}} & = \mathcal{D} (\boldsymbol{z}) =\text{Dec}_{\psi}(\boldsymbol{z}) \end{aligned}\end{align*}

where $\boldsymbol{\hat{x}}^{\text{tr}}$ is the reconstructed Tra. data, and $\text{MLP}_{\psi }$ is the multilayer perceptron for Tra. data reconstruction.

#### Topology discovery

To mitigate the adverse effects of modality bias phenomenon, MuST does not directly fuse the information of modalities, instead of measuring the importance of modality in Mor. and Tra. spot embedding space (Supplementary Fig. S1D). Specifically, we proposed a topology discovery strategy to accurately estimate global and local information of multi-modality data by extracting the topology from Mor./Tra. spot embedding space. The $E^{\text{tr}}$ and $E^{\text{mo}}$ are discovered with ($k$NN [59]) according to the distance in the Mor./Tra. spot embedding space.

#### Topology fusion loss

In addition, a topology fusion loss is proposed to minimize the topological differences between all modalities and the latent space with an adaptive motility’s importance estimated from the continuously updated Mor./Tra. ’s spot embedding:


(5) \begin{align*}& \begin{aligned} \mathcal{L}_{T}(\boldsymbol{X}, \boldsymbol{y}, \boldsymbol{z}) & = \sum_{\text{m}\in \mathcal{M}} \mathcal{L}_{T}^{\text{m}}(\boldsymbol{X}^{\text{m}}, E^{\text{m}},\boldsymbol{y}, \boldsymbol{z}) \\ \mathcal{L}_{T}^{\text{m}}(x^{\text{m}}, \boldsymbol{E}^{\text{m}}, \boldsymbol{y}, \boldsymbol{z}) & = \sum \{ \mathcal{T}_{ij}^{\text{m}} \log(S_{ij}) +(1-\mathcal{T}_{ij}^{\text{m}})\log(1-S_{ij}) \}, \\ \end{aligned}\end{align*}

where $\mathcal{M}$ is the set of modalities, in this paper, $\mathcal{M} = \{\text{tr}, \text{mo}\}$. $\boldsymbol{X}^{\text{m}}$ and $E^{\text{m}}$ are the data and topological structure of modality $\text{m}$. $S_{ij}$ is used to describe the connection strength between data $\boldsymbol{z}_{i}$ and $\boldsymbol{z}_{j}$:


(6) \begin{align*}& \begin{split} {S}_{ij} &= \kappa \left({\boldsymbol{z}}_{i}, {\boldsymbol{z}}_{j} \right)\!, \\ \kappa(a, b) &= (1 + || a - b ||^{2} / \nu)^{-\frac{\nu+1}{\nu}} \end{split}\end{align*}

where the kernel function $\kappa (a,b)$ maps the relationship of the input data to the similarity, and the degree of freedom $\nu $ is related to the number of the dimension. $\mathcal{T}_{ij}^{\mathbf{m}}$ is the topological priory between vector $\boldsymbol{y}_{i}^{\text{m}}$ and $\boldsymbol{y}_{j}^{\text{m}}$:


(7) \begin{align*}& \begin{split} {\mathcal{T}}_{ij}^{\text{m}} = \left[1 + \mathcal{H}_{ij}^{\text{m}}\left(e^\alpha-1\right)\right] \kappa(\boldsymbol{y}_{i}^{\text{m}}, \boldsymbol{y}_{j}^{\text{m}}) \end{split}\end{align*}

where $\mathcal{H}_{ij}^{\text{m}}$ is the augmentation relationship between $\boldsymbol{y}_{j}^{\text{m}}$ and $\boldsymbol{y}_{i}^{\text{m}}$ (defined in Equation (2)), and $\alpha $ is the hyper-parameter to control the influence of the contrastive loss in representation learning.

#### Overall loss function

Our overall loss function is formulated as follows:


(8) \begin{align*}& \mathcal{L} = \mathcal{L}_{T}(\boldsymbol{X}) + \lambda \mathcal{L}_{R}(\boldsymbol{x}^{\text{tr}}, \hat{\boldsymbol{x}}^{\text{tr}})\end{align*}

where $\lambda $ is a hyper-parameter tuning the influence between two loss functions. The reconstruction loss $\mathcal{L}_{R}(\boldsymbol{x}^{\text{tr}}, \hat{\boldsymbol{x}}^{\text{tr}})$ is designed to ensure that the $\mathcal{F}(\cdot )$ loses as little information about the gene modality as possible:


(9) \begin{align*}& \mathcal{L}_{R}(\boldsymbol{x}^{\text{tr}}, \hat{\boldsymbol{x}}^{\text{tr}}) = \frac{1}{N} \sqrt{\sum^{N}_{i=0}(\boldsymbol{x}^{\text{tr}}_{i} - \hat{\boldsymbol{x}}^{\text{tr}}_{i})^{2}}\end{align*}

where $\boldsymbol{x}^{\text{tr}}_{i}$ and $\hat{\boldsymbol{x}}^{\text{tr}}_{i}$ are the original and reconstructed Tra. data, respectively.

### Downstream tasks of MuST

The proposed MuST is dedicated to learning a unified embedding space, which is used for several downstream tasks. These downstream tasks are used in combination to confirm the results of ST analyses. It is important to note that, except for the spot visualization task, instead of designing entirely new methods for each downstream task, we apply specific techniques to MuST representations to achieve better results. The predefined downstream tasks of MuST are described below:

#### Spatial clustering

This task focuses on the clustering of spots based on the embedding space. It aids in identifying regions of interest or patterns that might be indicative of specific tissue structure or cell type. Spa. clustering accomplishes clustering according to a universal latent space, and various popular clustering methods can be employed. For fairness of comparison with the baseline methods, we show the clustering results of mclust [45] in our paper.

#### Spot visualization

An essential component of the embedding method, visualization allows researchers to intuitively understand and interpret the Spa. distribution and relationships between different points. The MuST ensures that the embedding space is accessible to different visualization techniques, providing clear and meaningful insights. Note that UMAP [47] and t-SNE [60] may produce false or overly sharp boundaries. We prioritize training an MLP network based on our topology preserving loss to map uniform embeddings onto a 2D visualization space [46]. For details,


(10) \begin{align*}& \begin{aligned} \boldsymbol{z}^{\text{vi}} & = \text{MLP}_{\phi^{\text{vi}}}(\boldsymbol{z}), & \boldsymbol{z}^{\text{vi}} \in \mathbb{R}^{2}, \end{aligned}\end{align*}

where $\boldsymbol{z}^{\text{vi}}$ is the visualization embedding of $\boldsymbol{z}$ and $\text{MLP}_{\phi ^{\text{vi}}}$ is the MLP for the visualization. The loss function is


(11) \begin{align*}& \begin{aligned} L_{\text{vi}}(\boldsymbol{z}) & = \sum \{ \mathcal{T}_{ij} \log(S_{ij}) +(1-\mathcal{T}_{ij})\log(1-S_{ij}) \}, \\ \mathcal{T}_{ij} & = \kappa(\boldsymbol{z}_{i}, \boldsymbol{z}_{j}), \;\;\; S_{ij} = \kappa \left({\boldsymbol{z}}^{\text{vi}}_{i}, {\boldsymbol{z}}^{\text{vi}}_{j} \right) \end{aligned}\end{align*}

where $\boldsymbol{z}_{i}$ and $\boldsymbol{z}_{j}$ are two spot data in the universal latent space $\boldsymbol{z}$, $\boldsymbol{z}_{i}^{\text{vi}}$, and $\boldsymbol{z}_{j}^{\text{vi}}$ are two spot data in the universal latent space $\boldsymbol{z}^{\text{vi}}$. The $\boldsymbol{z}_{j}$ is the augmented data of $\boldsymbol{z}_{i}$ or sampled from the same modality.

#### Spatial deconvolution

One of the critical challenges in ST is to ensure that the signals detected from a given spot are representative of a single-cell type or state, rather than a mixture of multiple sources. The purity of a spot in this context refers to the extent to which the observed Tra. profile can be attributed to a single cellular entity. Using the unified embedding space learned by MuST, the profile of each spot is compared with a library of known single-cell Tra. signatures. The similarity between the spot profile and the single-cell signatures provides an estimate of the purity of the spot. A high similarity to a single signature indicates high purity, while similarities to multiple signatures indicate potential mixing. MuST performs this task based on a unified embedding space without the need for reference information. For details, we use the mean vector as the standard vector for each cluster in the universal latent space. The Lasso [49] method is then used to determine the sparse weight of the data of each point characterized by the standard vector. The standard deviation of the sparse weight is used to estimate the impurity of the spot.

#### Marker gene analysis with deep learning interpretability

Given the often “black box” nature of deep learning models, MuST places a strong emphasis on interpretability. This task seeks to understand the model’s decision-making process, particularly in the context of identifying marker genes that may be indicative of specific cellular states or activities. In MuST, the amount by which changing a gene changes the position of the embedding in the universal latent space is used to define the importance of that gene. Specifically, changing an important gene will significantly change the position of the embedding of the data in the universal latent space.

#### Spatial trajectories inference

Understanding how cells differentiate and develop over time is crucial for many biological applications. MuST facilitates the inference of these trajectories, providing insights into developmental pathways, and potential regulatory mechanisms. MuST has integrated the Spa. position information into the unified embedding, so we directly use the PAGA [22] unified embedding method for Spa. trajectory inference.

### Performance evaluation protocol

We evaluate Spa. clustering performance using the adjusted rand index (ARI):


(12) \begin{align*}& \text{ARI} = \frac{\Sigma_{ij}\binom{n_{ij}}{2} - [\Sigma_{i}\binom{a_{i}}{2}\Sigma_{j}\binom{b_{j}}{2}] / \binom{n}{2}}{\frac{1}{2}[\Sigma_{i}\binom{a_{i}}{2}+\Sigma_{j}\binom{b_{j}}{2}] - [\Sigma_{i}\binom{a_{i}}{2}\Sigma_{j}\binom{b_{j}}{2}] / \binom{n}{2}}\end{align*}

where the ARI near 1 indicates a strong match to ground truth clustering, whereas values near 0 suggest random assignment. In the implementation, we use the adjusted_rand_score function from the scikit-learn Python package [61].

The mean relative rank error (MRRE) [62] measures the average of changes in neighbor ranking between the two spaces:


(13) \begin{align*}& \text{MRRE} = \frac{1} {(M \frac{|M-2 k|}{k})}\sum_{i=1}^{M} \sum_{j \in \mathcal{N}_{i,k}^{(l)}}\frac{|r^{(l)}_{i,j}-r^{(l^{\prime})}_{i,j}|}{r^{(l)}_{i,j}},\end{align*}

where $k$ is the sensing range of the metric, $r^{(l)}$ and $r^{(l^{\prime})}$ indicate the rank of data $j$ to data $i$ in the input space and the embedding space, $M$ is the number of the data. The rank is calculated by the Euclidean distance between the data. The $\mathcal{N}_{i,k}^{(l)}$ is the $k$ nearest neighbors of data $i$ in the input space.

The value used on gene expression analysis of the new region (Fig. 6G) on DLPFC slide 151 673 is calculated as follows:


(14) \begin{align*}& \bar{x_{i}} = \left( {\text{MEAN}_{i \in \mathcal{N}(i)}\left(\sum_{g \in \text{Gene}} {x^{\text{tr}}_{i, g}}\right)} \right)^{0.25}\end{align*}

where $x^{\text{tr}}_{i, g}$ represent the $g$ normalized genes on $i$th spot of Tra. input $x^{\text{tr}}$. The neighborhood of a spot $\mathcal{N}(i)$ is all spots of its cluster or itself.

### Data description

To evaluate the performance of MuST and the baseline methods in ST, we employ five tissues (eight datasets) as shown in Supplementary Table S1 corresponding to five subsections in the “Results” section.

- Two mouse embryos (E9.5 and E14.5) datasets are acquired using Stereo-seq data, which we download them from https://db.cngb.org/stomics/mosta/. The E9.5 embryo data consists of $5913$ bins and $25\,568$ genes, while the E14.5 embryo data consists of $92\,928$ bins and $18\,566$ genes.
- The Slide-seqV2 dataset acquired from mouse hippocampus and is downloaded from SCP354. We use the Puck_200115_08 section with $53\,172$ spots and $23\,264$ genes.
- The mouse brain tissue is downloaded from the publicly available 10$\times $ Genomics Data Repository https://www.10xgenomics.com/resources/datasets. This dataset has two sections, including a coronal mouse brain section and a mouse sagittal posterior brain section. The former contains $2702$ spots with $32\,285$ genes captured and is manually annotated with $52$ regions using the Allen Brain Atlas reference https://mouse.brain-map.org/static/atlas. The latter contains $3355$ spots with $32\,285$ genes captured and is manually annotated with $71$ regions using the Allen Brain Atlas reference.
- Two mouse olfactory bulb datasets are acquired using Stereo-seq data and Slidev2-seq data, which are further processed and annotated. The former data are downloaded from https://github.com/JinmiaoChenLab/SEDR_analyses. These data contain $19\,109$ spots and $27\,106$ genes. The latter data are downloaded from SCP815. These data contain $20\,139$ spots and $21\,220$ genes.
- The LIBD human dorsolateral prefrontal cortex (DLPFC) with $12$ tissue slices is acquired with 10$\times $ Visium. This dataset is available at http://research.libd.org/globus. The number of spots in each slice ranges from $3460$ to $4789$, with $33\,538$ genes captured. Each slice is manually annotated to contain five to seven regions, namely the DLPFC layers and WM.

### Comparison with baseline methods

To showcase the effectiveness of MuST in representation learning for ST data, we compare MuST with six state-of-the-art methods, including GraphST [15], STAGATE [25], DeepST [35], SpaGCN [31], MUSE [16], and stSME [33].

#### GraphST

GraphST is a method that integrates GNNs with self-supervised contrastive learning specifically for ST data, offering superior performance in Spa. clustering, multisample integration, and cell-type deconvolution, outpacing current methods across various tissue types and platforms. For DLPFC dataset, to reproduce the results in original paper as much as possible, we set the parameter “beta” to 0.5 and the dimension of the embedding layer to 72. For other datasets, we use the default parameters.

#### STAGATE

STAGATE is another deep learning model-based method that combines an auto-encoder with a graph attention mechanism to learn latent representation by modeling both gene expression profiles and Spa. location information. We ran STAGATE for Spa. clustering and vertical and horizontal ST data integration. All experiments are implemented using the recommended parameters in the package vignette. Specifically, with raw gene expressions, the top 3000 highly variable genes are first selected and then log-transformed and normalized according to library size. The parameter “alpha” was set to 0. The learning rate and training epoch are left at the default 0.0001 and 500, respectively.

#### DeepST

DeepST is an advanced deep learning framework for ST that outperforms existing methods in identifying Spa. domains, offering efficient data integration across batches or technologies, and showing adaptability for other Spa. omics data. For DLPFC dataset, we set the parameter “nbr_k” to 5, “tree_k” to 15, “adj_w” to 0.1, and “graph_conv” to ResGatedGraphConv. For other datasets, we use the default parameters.

#### SpaGCN

SpaGCN is a graph convolutional network approach that integrates gene expression, Spa. location information, and histological images for ST data analysis. SpaGCN is one of the only two other methods that can perform horizontal ST data integration. Following the tutorial, we applied SpaGCN to Spa. clustering and horizontal ST data integration with the default parameter settings. In particular, the parameter “histology” was set to “False”. The learning rate and max training epoch are set to 0.05 and 200, respectively.

#### MUSE

MUSE is a novel approach that integrates Mor. and spatially resolved Tra. data to uncover tissue subpopulations, compensates for modality-specific noise, and offers enhanced insights into cellular states and organization in both healthy and diseased tissues. We use the default parameters for all experiments.

#### stSME

stSME is a comprehensive Python software that employs innovative integrative analysis methods to harness Spa., Mor., and Tra. data from ST, enabling precise cell type identification, modeling of cellular evolution within tissues, and detection of hotspot regions for heightened cell-to-cell interactions in both healthy and diseased contexts. We use the default parameters for all experiments.

#### MuST

We use dropout to make learning process robust. For all experiments, the input dimension of Tra. modality is 3000 and the input dimension of Mor. modality is 500. For most of experiments, we set the dimension of hidden representation as 72. In information extraction, we employ a graph encoder for each modalities including a structure module (d/input_dimension, 72, 72, 72). To combine both modalities, we add them by element to get a joint representation. In information fusion, we employ an MLP for encoding including a structure module (72, 72) for most of datasets. In gene reconstruction, we employ a graph decoder including a structure module (d/72, gene_dimension).

We run all the experiments using a Ubuntu server and a single A100 GPU with 40 GB memory for 10$\times $ Visium platform and 80 GB memory for other platforms. To ensure that each method achieves its optimal performance, we use the grid search method to find the optimal hyperparameter. For baseline methods, we use the default hyperparameter setting according to the released code.

In the following, we discuss the function of different hyperparameters in MuST and propose typical value ranges. For Stereo-seq platform, the $K^{\text{tr}}$ adjusts the size of Hop-1 neighborhood for Tra. modality and the search space is typically set to $\{7, 9, 10\}$. The $r_{u}^{\text{tr}}$ adjusts the strength of augmentation which is typically set to $\{0.1, 1\}$. The $\nu $ adjusts the degree of freedom of kernel function which is typically set to $\{0.01, 0.05\}$ which is related to dimension. The $d_{\text{emb}}$ denotes the dimension of embedding which is typically set to $\{64, 72\}$. The $\lambda $ adjusts the effects of the reconstruction loss which is typically set to $\{0.0015, 0.001, 0.015\}$ according to dataset size. The $\theta $ is set to $\{0.1, 0.2, 1\}$ in accordance with dataset size. The $\tau $ denotes quantity of the minimum requirement of cells a gene is expressed which is typically set to ${50, 500}$.

For Slide-seq platform, the $K^{\text{tr}}$ and $K^{\text{mo}}$ adjusts the size of Hop-1 neighborhood for Tra. modality and the search space is typically set to $\{25, 50\}$. The $\nu $ adjusts the degree of freedom of kernel function which is typically set to $\{0.005, 1\}$ which is related to dimension. The $d_{\text{emb}}$ denotes the dimension of embedding which is typically set to $\{72, 100\}$. The $\lambda $ adjusts the effects of the reconstruction loss which is typically set to $\{0.00015, 0.005\}$ according to dataset size.

For 10$\times $ Visium platform, the $K^{\text{tr}}$ and $K^{\text{mo}}$ adjusts the size of Hop-1 neighborhood for Tra. modality and Mor. modality and the search space is typically set to $\{3, 5, 7, 9, 11\}$. The $r_{u}^{\text{tr}}$ and $r_{u}^{\text{mo}}$ adjusts the strength of augmentation for each modality which is typically set to $\{0.1, 0.3, 1\}$. The $\nu $ adjusts the degree of freedom of kernel function which is typically set to $\{0.05, 0.13, 0.15\}$ which is related to dimension. The $\lambda $ adjusts the effects of the reconstruction loss which is typically set to $\{0.001, 0.01, 0.12\}$ according to dataset size. The $\theta $ adjusts the proportion of Tra. feature which is typically set to $\{0.7, 0.9\}$. The $N_{\text{MLP}}$ adjusts the number of layers in fusion encoder which is typically set to $\{1, 3\}$. The $lr$ adjusts the learning rate which is typically set to $\{0.001, 0.0014\}$. We also apply refinement on clustering in specific to DLPFC dataset. We replace the label of each spot with the most occurring label in his 6-nearest neighborhood and itself.

### Statistics and reproducibility

The details about experimental design and statistics used in different data analyses performed in this study are given in the respective sections of results and methods.

**Key Points**

- **Modality Bias in Spatial Transcriptomics (ST):** ST data suffer from modality bias, where inconsistent contributions from Tra., Spa., and morphological modalities cause analysis methods to overly rely on the dominant modality, hindering balanced downstream performance.
- **Multiple-modality Structure Transformation (MuST) for Unified Multi-modality Representation:** The proposed method, MuST, addresses modality bias by integrating multi-modal information into a unified latent space. It leverages topology discovery and a topology fusion loss to align and harmonize structures across modalities.
- **Superior Accuracy and Biological Relevance:** MuST achieves state-of-the-art performance in identifying tissue structures and biomarkers, demonstrating both high analytical precision and strong biological interpretability across diverse downstream tasks.

## Supplementary Material

main_bib_supp_bbaf405

## Data Availability

We use publicly available datasets in this study (details in the Section Data description). To make the results presented in this study reproducible, all processed data are available in Single Cell Portal SCP2425 (the access permission will be removed upon acceptance).

## Code availability

The MuST software package, implemented in Pytorch, is available free from https://github.com/zangzelin/MuST (the access permission will be removed upon acceptance), and as a Supplementary Software 1 accompanying this manuscript.
