# Supplementary material for: MuST: multiple-modality structure transformation for single-cell spatial transcriptomics
Source: Brief Bioinform. 2025 Aug 28;26(4):bbaf405. doi: 10.1093/bib/bbaf405 (PMC12392272; doi:10.1093/bib/bbaf405)
Supplement: main_bib_supp_bbaf405 [file main_bib_supp_bbaf405.pdf]

Comparing the modality bias phenomenon on input data and MuST representations

We use the Shaply value [42] between ground truth and input data/MuST embeddings to evaluate the contributions of transcriptomic (Tra.) modality, morphology (Mor.) modality and location (Loc.) modality. The results show that MuST greatly alleviates the modality bias phenomenon in ST data. In terms of statistical evidence, the contribution Tra. modality, Mor. modality and Loc. modality are more balanced on MuST embeddings (Fig. S2B and Fig. S3A). In addition, Tra. modality and Mor. modality play different role in terms of tissue structure discovery (Fig. S2C and Fig. S3B).

Ablation study

Table S5 presents the results of an ablation study evaluating MuST's performance on the mouse sagittal posterior brain section. This study aims to assess the impact of excluding various components from the MuST model—specifically, the morphological modality topology loss ($\mathcal{L}_T^{mo}$), topology fusion loss ($\mathcal{L}_T$), and Graph Neural Network (GNN)—on the performance metrics: MRRE^{tr}, MRRE^{mo}, and MRRE$^{tr\&mo}$.

Our findings demonstrate that the complete MuST model achieves superior performance, with MRRE^{mo} recorded at 0.051 and MRRE$^{tr\&mo}$ at 0.079. The elimination of $\mathcal{L}_T^{mo}$ results in a negligible increase in MRRE^{mo} to 0.054, indicating that exclusive reliance on transcriptomic modality information is inadequate for capturing the morphological modality's topology.

Further removal of $\mathcal{L}_T$ leads to a significant performance decline, with MRRE$^{tr\&mo}$ rising to 0.121, underscoring the essential role of $\mathcal{L}_T$ in maintaining the data's topological structure. The most considerable performance degradation is observed when both $\mathcal{L}_T$ and the GNN are excluded, elevating MRRE$^{tr\&mo}$ to 0.124. This suggests that integrating spatial location information is crucial for preserving the topological structure.

Interestingly, when $\mathcal{L}_T^{tr}$ is removed, MRRE^{tr} decreases to 0.024, possibly due to the model overfitting to the transcriptomic modality. This result deviates from our objective of utilizing multimodal information to enhance overall model performance.

These observations confirm that the complete MuST model performs optimally, and the stepwise removal of its components progressively degrades performance. Thus, each component's contribution is critical to achieving the best results.

Comparing Leiden, Louvain, and mclust clustering on MuST representations

In MuST, we chose mclust as the default clustering method because our assessment shows that mclust performs better than Leiden and Louvain in most cases. Here, we compare the clustering results on different datasets using Leiden, Louvain, and mclust shown in Fig. S14. mclust consistently outperforms Leiden and Louvain on all datastes. Visually, the clusters identified by mclust are more continuous. Nevertheless, we also include Leiden and Louvain in MuST as alternative clustering methods.

Supplementary Tables

Table S1. Description of all ST datasets used in this study.
Table S2. The performance comparison on MRRE across four tissues. The baseline methods are MUSE, SpaceGCN, and GraphST. The MRRE metric measures the discrepancy between marked areas and anatomical regions, and a lower MRRE indicates better performance. The average value is computed from ME9.5 to 151508.
Table S3. The performance comparison on ARI across 12 slices of DLPFC. The baseline methods are MUSE, SpaceGCN, and GraphST. The ARI metric measures the spatial clustering performance, and a higher ARI indicates better performance.
Table S4. Hyperparameters of MuST in all ST datasets.
Table S5. Ablation study on MuST performance for mouse sagittal posterior brain section. The metrics MRRE^{tr}, MRRE^{mo} and MRRE$^{tr\&mo}$ define the input space as Tra. modality, Mor. modality and both combined, respectively.
Table S6. Marker gene selection of MuST on mouse embryo at E14.5 dataset acquired with Stereo-seq.
Table S7. Marker gene selection of MuST on mouse embryo at E9.5 dataset acquired with Stereo-seq.
Table S8. Marker gene selection of MuST on mouse hippocampus dataset acquired with Slide-seqV2.
Table S9. Marker gene selection of MuST on coronal mouse brain section acquired with 10x Visium.
Table S10. Marker gene selection of MuST on mouse sagittal posterior brain section acquired with 10x Visium.
Table S11. Marker gene selection of MuST on mouse olfactory bulb tissue dataset acquired with Stereo-seq.
Table S12. Marker gene selection of MuST on mouse olfactory bulb tissue dataset acquired with Slide-seqV2.
Table S13. Marker gene selection of MuST on slice 151673 of DLPFC dataset acquired with 10x Visium.

Supplementary Figures

Figure S1. MuST alleviates the modality bias phenomenon in mouse brain data. **A** H&E image of mouse brain coronal section and mouse sagittal posterior brain section. **B** Contribution of the morphology (Mor.) modality, transcriptome (Tra.) modality and spatial (Spa.) modality input data or MuST embedding to data labeling. **C** Contribution visualization of the Mor. modality, Tra. modality and Spa. modality input data or MuST embedding to data labeling.
Figure S2. MuST alleviates the modality bias phenomenon in mouse embryo data. **A** Contribution of the morphology (Mor.) modality, transcriptome (Tra.) modality and spatial (Spa.) modality input data or MuST embedding to data labeling on mouse embryo 14.5 and 9.5 datasets. **C** Contribution visualization of the Mor. modality, Tra. modality and Spa. modality input data or MuST embedding to data labeling.
Figure S3. MuST enables accurate identification of different organs in the Stereo-seq mouse embryo. **A** Tissue domain annotations of the E14.5 mouse embryo data obtained from the original Stereo-seq study. **B** Clustering results by GraphST and MuST on the E14.5 mouse embryo. **C** Cluster visualization of selected spatial domains identified by the original Stereo-seq study, MuST and GraphST, respectively.
Figure S4. MuST enables accurate identification of different organs in the Stereo-seq mouse embryo. **A** Tissue domain annotations of the E9.5 mouse embryo data obtained from the original Stereo-seq study. **B** Clustering results by GraphST and

MuST on the E9.5 mouse embryo. **C** Cluster visualization of selected spatial domains identified by the original Stereo-seq study, MuST and GraphST, respectively.

Figure S5. Spatial deconvolution of MuST's results in adult mouse brain section profiled by 10x Visium. **A** Allen Brain Institute reference atlas diagram and H&E image of the mouse cortex. **B** Allen Brain Institute reference atlas diagram and H&E image of the mouse sagittal. **C** Spatial deconvolution of the identified domains by MuST on coronal mouse brain section. **D** Spatial deconvolution of the identified domains by MuST on mouse sagittal posterior brain section. **E** Spatial deconvolution of the coronal mouse brain section. **F** Spatial deconvolution of the mouse sagittal posterior brain section.

Figure S6. Embedding analysis of MuST on the mouse olfactory bulb tissue and mouse embryo. **A** Mean relative rank error (MRRE) scores and UMAP visualizations generated by SpcGCN, MUSE, DeepST, GraphST and MuST representations on the Stereo-seq E14.5 mouse embryo data. Among them, methods MUSE and DeepST have an out of memory (OOM) problem. **B** MRRE scores and UMAP visualizations generated by the representations of five methods on the Stereo-seq E9.5 mouse embryo data. **C** MRRE scores, UMAP visualizations and PAGA graphs generated by the representations of five methods on the Stereo-seq mouse olfactory bulb tissue section. **D** MRRE scores, spot visualizations and PAGA graphs generated by the representations of five methods on the Slide-seqv2 mouse olfactory bulb tissue section.

Figure S7. Manual annotations and comparison of spatial domains identified by stSME, SpaGCN, MUSE, DeepST, GraphST and MuST on the 12 slices of DLPFC dataset.

Figure S8. Marker genes selection for each anatomical region of mouse hippocampus data acquired with SlideseqV2.

Figure S9. Marker genes selection for each region of coronal mouse brain section acquired with 10x Visium.

Figure S10. Marker genes selection for each region of mouse sagittal posterior brain section acquired with 10x Visium.

Figure S11. Marker genes selection for each laminar organization of coronal mouse olfactory bulb tissue datasets acquired with Slide-seqV2.

Figure S12. Marker genes selection for each laminar organization of coronal mouse olfactory bulb tissue datasets acquired with Stereo-seq.

Figure S13. Comparison analysis between Leiden, Louvain, and mclust with the output of MuST as input. Visualization of clustering results from Louvain, Leiden, and mclust on Stereo-seq E14.5 mouse embryo data (A), Stereo-seq 9.5 mouse embryo data (B), Stereo-seq mouse olfactory bulb tissue sections (C), Slide-seqV2 mouse olfactory bulb tissue sections (D), 10x Visium coronal mouse brain section (E), 10x Visium mouse sagittal posterior brain section (F), and SlideseqV2 mouse hippocampus data (G).

Table S1. Description of all ST datasets used in this study.

Platform	Tissue	Section	#Spots/bins	#Genes	Related figures	Reference
Stereo-Seq	Mouse embyro	E14.5	92928	18582	Fig. 2, Fig. C1, Fig. C4, Fig. C11, Tab. C3	[10]
		E9.5	5913	25568	Fig. 2, Fig. C2, Fig. C4, Fig. C11, Tab. C4	[10]
	Mouse olfactory bulb	MOB	19109	27106	Fig. 5, Fig. C4, Fig. C9, Fig. C11, Tab. C8	[10]
Slide-SeqV2	Mouse hippocampus	Puck_200115_08	53172	23264	Fig. 3, Fig. C6, Fig. C11, Tab. C5	[8]
	Mouse olfactory bulb	Puck_200127_15	20139	21220	Fig. 5, Fig. C4, Fig. C10, Fig. C11, Tab. C9	[8]
10x Visium	Mouse brain	Mouse Brain Section (Coronal)	2702	32285	Fig. 4, Fig. C3, Fig. C7, Fig. C11, Tab. C6	[49]
	Mouse brain	Mouse Brain Section (Sagittal-Posterior)	3355	32285	Fig. 4, Fig. C3, Fig. C8, Fig. C11, Tab. C7	
	Human dorsolateral prefrontal cortex (DLPFC)	151507	4220	33538	Fig. 6, Fig. C5	[41]
		151508	4381	33538	Fig. 6, Fig. C5	[41]
		151509	4788	33538	Fig. 6, Fig. C5	[41]
		151510	4595	33538	Fig. 6, Fig. C5	[41]
		151669	3636	33538	Fig. 6, Fig. C5	[41]
		151670	3484	33538	Fig. 6, Fig. C5	[41]
		151671	4093	33538	Fig. 6, Fig. C5	[41]
		151672	3888	33538	Fig. 6, Fig. C5	[41]
		151673	3611	33538	Fig. 6, Fig. C5, Tab. C10	[41]
		151674	3635	33538	Fig. 6, Fig. C5	[41]
		151675	3566	33538	Fig. 6, Fig. C5	[41]
		151676	3431	33538	Fig. 6, Fig. C5	[41]

Table S2. The performance comparison on MRRE across four tissues. The baseline methods are MUSE, SpaceGCN, and GraphST. The MRRE metric measures the discrepancy between marked areas and anatomical regions, and a lower MRRE indicates better performance. The average value is computed from ME9.5 to 151508.

Dataset	Mouse Hippocampus	Mouse Embryos		Mouse Brain	Mouse Olfactory Bulb		DLPFC				Average
	Slide-seqV2	ME14.5	ME9.5	posterior	Stereo-seq	Slide-seqV2	151672	151676	151670	151508	
MUSE	—	—	0.29	0.14	0.81	0.72	0.45	0.50	0.45	0.48	0.48
SpaceGCN	0.23	0.27	0.12	0.21	0.62	0.20	0.82	0.43	0.75	0.91	0.50
DeepST	—	—	0.23	0.12	0.40	0.34	0.58	0.40	0.56	0.59	0.40
GraphST	0.45	0.33	0.18	0.13	0.67	0.30	0.50	0.37	0.52	0.61	0.41
**MuST**	**0.13**	**0.15**	**0.10**	**0.08**	**0.19**	**0.16**	**0.42**	**0.31**	**0.40**	**0.41**	**0.26**

Table S3. The performance comparison on ARI across 12 slices of DLPFC. The baseline methods are MUSE, SpaceGCN, and GraphST. The ARI metric measures the spatial clustering performance, and a higher ARI indicates better performance.

Dataset	DLPFC												Average
	151676	151675	151674	151673	151672	151671	151670	151669	151510	151509	151508	151507	
MUSE	0.16	0.19	0.23	0.24	0.18	0.14	0.12	0.14	0.15	0.19	0.12	0.14	0.17
SpaceGCN	0.39	0.37	0.53	0.48	0.32	0.45	0.26	0.36	0.43	0.29	0.40	0.39	0.39
DeepST	0.46	0.53	0.51	0.59	0.41	0.54	0.33	0.34	0.35	0.40	0.38	0.52	0.45
GraphST	0.57	**0.62**	0.63	0.63	**0.63**	**0.63**	0.59	0.50	**0.51**	**0.42**	**0.51**	0.59	**0.57**
**MuST**	**0.64**	0.59	**0.65**	**0.65**	**0.63**	0.62	**0.65**	**0.61**	0.40	0.34	0.51	0.38	**0.57**

Table S4. Hyperparameters of MuST in all ST datasets.

Section	K^{mo}	K^{tr}	r_u^{mo}	r_u^{tr}	ν	d_{emb}	θ	λ
ME95	5	9	0.1	1	0.05	72	1	0.015
ME145	5	7	0.1	1	0.05	72	1	0.0015
MOB	10	10	0.1	0.1	0.01	64	1	0.001
DLPFC	3	11	1	0.3	0.13	72	0.9	0.12
Mouse Brain Section (Coronal)	7	7	0.1	0.1	0.05	72	0.9	0.01
Mouse Brain Section (Sagittal-Posterior)	5	9	1	0.1	0.15	72	0.7	0.001
Puck_200115_08	5	25	0.1	0.5	1	72	1	0.005
Puck_200127_15	10	50	0.1	0.5	0.005	100	1	0.00015

Table S5. Ablation study on MuST performance for mouse sagittal posterior brain section. The metrics MRRE^{tr}, MRRE^{mo} and $\text{MRRE}^{tr\&mo}$ define the input space as Tra. modality, Mor. modality and both combined, respectively.

Loss/Architecture Setting				MRRE		
$\mathcal{L}_T^{\text{tr}}$	$\mathcal{L}_T^{\text{mo}}$	$\mathcal{L}_T$	with GNN	**Tra.**	**Mor.**	**Tra. & Mor.**
✓	×	✓	✓	0.026	0.054	0.079
✓	×	×	✓	0.043	0.077	0.121
✓	×	×	×	0.024	0.100	0.124
✓	✓	✓	✓	0.027	0.051	0.079

Table S6. Marker gene selection of MuST on mouse embryo at E14.5 dataset acquired with Stereo-seq.

Clusters	Marker genes
C1	Afp, Hbb-bs, Tuba1a, Ttn, Hbb-bt, Apoa2, Mt1, H19, Col3a1, Col1a2, Lgals1, Fabp7
C2	Tuba1a, Fabp7, H19, Col3a1, Hbb-bs, Lgals1, Col1a2, Afp, Stmn2, Hbb-bt, Rtn1, Dbi
C3	Col3a1, Tuba1a, Col1a2, Ttn, Hbb-bs, Afp, Myh3, Gphn, Mt1, Hbb-bt, Krt5, Col1a1
C4	Afp, Apoa2, Slc25a37, Hbb-bt, Gpx1, Alb, Mt1, Apoa1, Hba-a2, Mt2, Col3a1, Hba-a1
C5	Krt5, Tuba1a, Krt15, Mt1, Hbb-bs, Afp, Col3a1, H19, Hbb-bt, Lgals1, Col1a2, Fabp7
C6	Ttn, Myh3, Neb, Hbb-bs, Tceal7, Actc1, Myl1, Nrk, Tnni2, Hba-a1, Col3a1, Acta1
C7	Ugt2a2, H19, Tuba1a, Col3a1, Sult1e1, Lgals1, Hbb-bs, Afp, Hbb-bt, Col1a2, Fabp7, Igf2
C8	Krt5, Cxcl14, Tuba1a, Afp, Perp, Col3a1, Anxa2, Hbb-bs, H19, S100a6, Krt15, Col1a2
C9	Col11a1, Col2a1, Tuba1a, Gphn, Hbb-bs, Col3a1, Afp, Malat1, H19, Hbb-bt, Mt1, Col1a2
C10	Fabp7, Tuba1a, Slc1a3, Dbi, Hbb-bs, Mylpf, Tubb2b, Spon1, H19, Fez1, Camk1d, Afp
C11	Trps1, Alx1, Mt1, Hbb-bs, Hpse2, Tuba1a, Col2a1, Camk1d, Malat1, Msx1, Col3a1, Afp
C12	Myh11, Acta2, Mylk, Tuba1a, Actg2, Hbb-bs, Hba-a1, Afp, Prkg1, H19, Col3a1, Fabp7
C13	Tuba1a, Map1b, Tmsb10, Col1a2, Col3a1, Hbb-bs, Nnat, Rtn1, H19, Camk1d, Afp, Lgals1
C14	Neurod6, Tuba1a, H19, Hbb-bs, Lgals1, Fabp7, Pantr1, Hbb-bt, Actc1, Nfib, Col3a1, Igf2
C15	Tuba1a, Rtn1, Hbb-bs, Stmn2, Map1b, H19, Gphn, Gap43, Tubb2a, Tmsb10, Lgals1, Afp
C16	Nppa, Myh6, Tuba1a, Tnnt2, Tnni3, H19, Hbb-bs, Afp, Hbb-bt, Actc1, Col3a1, Myl7

Table S7. Marker gene selection of MuST on mouse embryo at E9.5 dataset acquired with Stereo-seq.

Clusters	Marker genes
C1	Postn, Fabp7, Igf2, Hbb-bs, H19, Pantr1, Msx1, Gata6, Sox2, Ckb, Hbb-y, Igfbp2
C2	Fabp7, Igf2, Hbb-y, Hbb-bh1, Hoxb3os, Ckb, Pantr1, Col3a1, H19, Tmsb4x, Otx2, Cnn2
C3	Myl7, Actc1, Acta1, Tnni1, Myl4, Igf2, Fabp7, Tnnt2, Myh7, Hbb-y, Marcks, Lars2
C4	Fabp7, Igf2, Hbb-bh1, Ckb, H19, Hbb-y, Dlk1, Col3a1, Tmsb4x, Crabp1, Rfx4, Tuba1a
C5	Igf2, Fabp7, Myl7, Ckb, Zic1, Mest, H19, Crabp1, Foxf1, Peg3, Hbb-y, Actc1
C6	Fabp7, Igf2, Ckb, Hbb-bh1, H19, Hbb-y, Cnn2, Pantr1, Col3a1, Rfx4, Sox2, Vim
C7	Lars2, Fabp7, Marcks, Igf2, Cnn2, Flrt2, Zc3h7a, Ckb, Ebf1, Gm26561, Sulf1, Acta1
C8	Meox1, Myl7, Fabp7, Igf2, Msx1, Dmrt2, Mylpf, Acta1, Lars2, Actc1, Zc3h7a, Pantr1
C9	Fabp7, Igf2, Pantr1, Sox2, Id4, Hbb-y, Ckb, Rfx4, H19, Dlk1, Col3a1, Cnn2
C10	Fabp7, Igf2, Vim, Ckb, Hbb-y, Pantr1, Rfx4, Sox2, H19, Msi1, Lars2, Marcks
C11	Cpox, Hbb-bt, Fabp7, Mt2, Igf2, H19, Hba-a2, Mt1, Hbb-y, Sox2, Abcb10, Pantr1
C12	Fabp7, Igf2, Hbb-y, Ckb, H19, Crabp1, Pantr1, Zic1, Hbb-bh1, Sox2, Myl7, Vim
C13	Mylpf, Fabp7, Igf2, Pantr1, Hba-x, Hbb-bh1, Vim, Myl7, Meox2, Ckb, H19, Nnat
C14	Igf2, Fabp7, Ckb, Hbb-bh1, Hbb-y, H19, Ntn1, Msx1, Tmsb4x, Mest, Nkx6-1, Col3a1
C15	Fabp7, Igf2, Msx1, H19, Pantr1, Acta1, Hand2, Rfx4, Actc1, Tnni1, Dlk1, Myl7
C16	Hbb-y, Fabp7, Igf2, Sox2, H19, Gm42418, Ckb, Pantr1, Bpgm, Mt2, Cenpa, Ldha
C17	Fabp7, Igf2, H19, Myl7, Hbb-bs, Ckb, Pantr1, Dlk1, Sox2, Postn, Mest, Hoxc9
C18	Pax1, Fabp7, Arg1, Meox2, Vim, Igf2, Ckb, Sox2, Myl7, Pantr1, H19, Vcan
C19	Fabp7, Igf2, Ebf1, Pantr1, Ckb, Stmn2, Hbb-y, H19, Hbb-bh1, Lars2, Nefm, Vim
C20	Afp, Fabp7, Igf2, Pantr1, Ckb, H19, Myl7, Hbb-y, Sox2, Lars2, Actc1, Vim
C21	Fabp7, Hbb-bh1, Igf2, Hbb-y, Hoxb3os, Col3a1, Pantr1, Ckb, Tmsb4x, H19, Crabp1, Lars2
C22	Igf2, Fabp7, Ckb, Hbb-y, H19, Ntn1, Hbb-bh1, Col3a1, Rfx4, Cnn2, Pantr1, Tmsb4x

Table S8. Marker gene selection of MuST on mouse hippocampus dataset acquired with Slide-seqV2.

Clusters	Marker genes
C1	Plp1, Pcp4, Snap25, Mbp, Meg3, Nrgn, Hpca, Ncald, Mef2c, Mobp, Ttr, Ppp3ca
C2	Pcp4, Hpca, Nrgn, Plp1, Rora, Pcp4l1, Mbp, Ccdc136, Cit, Ttr, Tcf7l2, Tnnt1
C3	Nrgn, Snap25, Mef2c, Pcp4, Ncald, Hpca, Plp1, Meg3, Lamp5, Pde1a, 3110035E14Rik, Lmo4
C4	Plp1, Mbp, Pcp4, Mobp, Hpca, Qdpr, Mal, Cnp, Cldn11, Nrgn, Sept4, Ptgds
C5 (CA1)	Hpca, Ppp3ca, Nrgn, Itpka, Wipf3, Prkcb, Plp1, Tmsb4x, Pcp4, Cck, Meg3, Mbp
C6 (CA3)	Hpca, Cpne6, Ppp3ca, Cplx2, Snap25, Plp1, Chgb, Hs3st4, Nrgn, Pcp4, Calm2, Tmsb4x
C7 (Dentate Gyrus)	Hpca, Ppp3ca, Ncdn, Nrgn, Plp1, Cplx2, Snap25, Pcp4, Olfm1, Mbp, Itpka, Meg3
C8 (Interneuron)	Gad1, Sst, Pcp4, Plp1, Hpca, Mbp, Gad2, Nrgn, Cnr1, Snap25, Meg3, Ncald
C9	Meg3, Nrgn, Snhg11, Pcp4, Snap25, Hpca, Plp1, Malat1, Calb2, Ptk2b, Mef2c, Tmsb4x
C10 (LH)	Pcp4, Nrgn, Hpca, Plp1, Ptgds, Nwd2, Snap25, Nnat, Nefm, Ttr, Gap43, Meg3
C11 (V3)	Ttr, Nrgn, Pcp4, Plp1, 1500015O10Rik, Hpca, Zic1, Pcp4l1, Malat1, Mbp, Meg3, Enpp2
C12 (MH)	Nwd2, Zic1, Ttr, Hpca, Calb2, Pcp4, Tac2, Cd63, Plp1, Tcf7l2, Meg3, Gng8
C13	Apod, Ptgds, Aqp4, Agt, Pcp4, Vim, Hpca, Plp1, Nrgn, Zic1, Nwd2, Ttr

Table S9. Marker gene selection of MuST on coronal mouse brain section acquired with 10x Visium.

Clusters	Marker genes
C1	Tcf7l2, Nrgn, Sparc, Ddn, Ctxn3, Camk2n1, Slc17a6, Slc17a7, Hlf, Ctxn1, Rims3, Adarb1
C2	Nrgn, Mobp, Tcf7l2, Slc17a7, Mbp, Pvalb, Sparc, Prkcd, Vxn, Camk2n1, Fxyd7, Ddn
C3	Hap1, Baiap3, Pmch, 6330403K07Rik, Slc17a7, Nrgn, Camk2n1, Resp18, Nsmf, Tcf7l2, Ddn, Cartpt
C4 (V3)	Slc17a7, Mia, Ncdn, Thy1, Camk2n1, Hap1, Cck, Mbp, Nrgn, Baiap3, Tcf7l2, Nsmf
C5	Ddn, Ttr, Camk2n1, Fth1, Hpca, Nrgn, Slc17a7, Mbp, Spink8, Pantr1, Cabp7, Ctxn1
C6 (Dentate Gyrus)	Camk2n1, Slc17a7, Nrgn, Vxn, Tcf7l2, Eef1a1, Pcp4, Ddn, Hpca, Nsmf, Mbp, Adcy1
C7	Syn2, Camk2n1, Nptxr, C1ql3, Nr2f2, Vxn, Ddn, Nnat, Uchl1, Nrgn, Lypd1, Pcp4
C8	Prkcd, Adarb1, Tcf7l2, Nrgn, Ctxn1, Rora, Tnnt1, Pcp4, Atp2b1, Slc17a7, Ptpn3, Mobp
C9 (Cerebral cortex 2)	Camk2n1, Nrgn, Mef2c, Snap25, Dkkl1, Mbp, Plcxd2, Slc17a7, Atp1a1, Pcp4, Ddn, Vxn
C10	Camk2n1, Ddn, Slc17a7, Mbp, Penk, Hpcal4, Hpcal1, Hpca, Fth1, Syn2, Ctxn1, Vxn
C11	Slc17a7, Nrgn, Tcf7l2, Camk2n1, Sparc, Ddn, Baiap3, Nnat, Rpl17, Hap1, Tmsb4x, Thy1
C12	Camk2n1, Ddn, Mbp, Olfm1, Hpca, Nrgn, Slc17a7, Nptxr, Penk, Ctxn1, Hpcal4, Vxn
C13	Penk, Slc17a7, Camk2n1, Ctxn1, Arpp21, Hpcal4, Nrgn, Ddn, Tcf7l2, Mbp, Wfs1, Eef1a1
C14 (Cerebral cortex 1)	Camk2n1, Mbp, Pcp4, Nrgn, Lamp5, Tubb2a, Cplx2, Tmsb10, Ddn, Atp1a1, Slc17a7, Vxn
C15 (Cerebral cortex 3)	Camk2n1, Slc17a7, Vxn, Eef1a1, Ddn, Tcf7l2, Mbp, Nrgn, 1110008P14Rik, Fth1, Nptxr, Ttr
C16 (Fiber tracts)	Mbp, Fth1, Mobp, Thy1, Ncdn, Camk2n1, Tcf7l2, Nrgn, Tmsb4x, Slc17a7, Snap25, Cnp
C17	Prkcd, Adarb1, Tcf7l2, Rora, Atp2b1, Pcp4, Nrgn, Tnnt1, Camk2n1, Ctxn1, Slc17a7, Ptpn3
C18 (CA1)	Hpca, Camk2n1, Nrgn, Tmsb4x, Wipf3, Slc17a7, Vxn, Spink8, Tcf7l2, Ddn, Mbp, Ppp3ca
C19 (Cerebral cortex 4)	Vxn, Tmsb4x, Slc17a7, Camk2n1, Mbp, Diras2, Ttc9b, Mobp, Nr4a2, Nptxr, Ncald, Nrgn
C20 (CA3)	Camk2n1, Slc17a7, Tcf7l2, Nrgn, Vxn, Hpca, Ctxn1, Mbp, Ddn, Fth1, Hpcal4, Cnih2

Table S10. Marker gene selection of MuST on the mouse sagittal posterior brain section acquired with 10x Visium.

Clusters	Marker genes
C1 (CA1)	Mbp, Pcp2, Camk2a, Nrgn, Hpca, Ddn, Rasgrp1, Cck, Nnat, Stmn1, Pvalb, Ctxn1
C2	Nrgn, Mbp, Ddn, Camk2a, Pcp4, Slc1a2, Pvalb, Ctxn1, Pcp2, Plp1, Pantr1, Snap25
C3	Mbp, Camk2a, Cpe, Rasgrp1, Plp1, Atp1a1, Nnat, Ctxn1, Slc17a7, Snap25, Nrgn, Cck
C4	Nnat, Ccn3, Ly6h, Mbp, Dcn, Crym, Nptxr, Ctxn1, Ttr, Ddn, Trh, Pcp2
C5 (Coronal structure)	Atp1a1, Camk2a, Grin2c, Nrep, Adcy1, Mbp, Pcp2, Cbln3, Pvalb, Cbln1, Gabra6, Nrgn
C6 (Fiber tracts)	Mbp, Plp1, Nrgn, Cpe, Cck, Atp1a1, Camk2a, Trf, Mobp, Pcp2, Camkv, Ctxn1
C7 (Cerebral cortex 2)	Pcp2, Mbp, Nrgn, Atp1a1, Camk2n1, Pcp4, Camk2a, Slc1a2, 1110008P14Rik, Nnat, Dkk3, Ddn
C8	Mbp, Cpe, Pvalb, Nefl, Mobp, Snap25, Pcp2, Vamp1, Camk2a, Nrgn, Cck, Hoxb5
C9 (Dentate Gyrus)	Mbp, Pcp2, Ddn, Camk2a, Olfm1, Pvalb, Nrgn, Synpr, Snhg11, Ctxn1, Ncdn, Pcp4
C10 (Thin layer 1)	Pcp2, Mbp, Pvalb, Atp1a1, Itpr1, Gng13, Camk2a, Mobp, Stmn1, Cbln1, Car8, Cbln3
C11 (Thin layer 2)	Pcp2, Mbp, Nptx1, Pvalb, Nrgn, Cbln1, Calm2, Ppp1r17, Car8, Gabra6, Camk2a, Pcp4
C12 (Thin layer 3)	Pcp2, Mbp, Cbln1, Pvalb, Atp1a1, Mobp, Camk2a, Nptx1, Car8, Gabra6, Cbln3, Calm2
C13 (Cervical)	Hoxb5, Mbp, Pcp2, Cck, Pvalb, Camk2a, Slc6a5, Nefm, Vamp1, Nrgn, Zwint, Vsnl1
C14	Mbp, Camk2n1, Camk2a, Mef2c, Nnat, Cck, Ctxn1, Pcp2, Lypd1, Stx1a, Atp1a1, Snhg11
C15 (Cerebral cortex 1)	Prkcd, Tnnt1, Cck, Pcp2, Rasgrp1, Ntng1, Mbp, Rgs16, Tcf7l2, Pdp1, Nrgn, Ndrg4
C16	Ddn, Pvalb, Camk2a, Mbp, Nrgn, Vsnl1, C1ql2, Uchl1, Atp1a1, Pcp2, Ctxn1, Stmn1
C17	Mbp, Pcp2, Nrgn, Camk2a, Pvalb, Igfbp2, Nnat, Ddn, Cck, Ctxn1, Atp1a1, Ccn3
C18	Mbp, Nptxr, Trh, Pcp2, Nrgn, Ctxn1, Nnat, Camk2a, Ccn3, Lypd1, Hap1, Ddn
C19	Lypd1, Ctxn1, Pgrmc1, Mbp, Nnat, Ccn3, Ttr, Nr2f2, Pcp2, Ptgds, Ly6h, Trh
C20 (CA3)	Crym, Hpca, Pvalb, Mbp, Camk2a, Nrgn, Cpne6, Ccn3, Pcp2, Ctxn1, Ak5, Cnih2

Table S11. Marker gene selection of MuST on mouse olfactory bulb tissue dataset acquired with Stereo-seq.

Clusters	Marker genes
ONL	Pcp4, Gad1, Gng13, Fabp7, Ptn, S100a5, Calb2, Nrxn3, Apoe, Kcnb2, Ptgds, Ptprd
EPL	Calb2, Nrsn1, Pcp4, Nxph1, Nrxn3, Apoe, Gng13, Fabp7, Ptn, S100a5, Cck, Zic1
GCL	Gad1, Pcp4, Pcp4l1, Nme7, Ncdn, Ptprd, Stxbp6, Spp1, Gabra1, Atp1b1, Prkca
IPL	Spp1, Stmn2, Uchl1, Gad1, Gabra1, Pcp4, Cpe, Lhfpl3, Nmb, Nef, Ptprd, Cplx1
MCL	Gad1, Pcp4, Nrxn3, Kcnb2, Ppp3ca, Atp1a2, Plp1, Calb2, Nme7, Pbx3, Tenm3, Cpne4
GL	Gng13, Ptn, Fabp7, Ptgds, S100a5, Apoe, Atp1a2, Npy, Plp1, Gad1, Pcp4, Snap25
RMS	Plp1, Pcp4, Nrep, Gad1, Nme7, Nrxn3, Pbx3, Mobp, Ptgds, Apoe, Tenm3, Calb2

* Here we assign clusters to the corresponding tissue structure: olfactory nerve layer (ONL), external plexiform layer (EPL), granule cell layer (GCL), internal plexiform layer (IPL), mitral cell layer (MCL), glomerular layer (GL), and rostral migratory stream (RMS) according to the ground truth.

Table S12. Marker gene selection of MuST on mouse olfactory bulb tissue dataset acquired with Slide-seqV2.

Clusters	Marker genes
GCL	Pcp4, Nrxn3, Camk2b, Doc2g, Meis2, Ppp3ca, Nrsn1, S100a5, Cck, Fabp7, Atp1a2, Dlg2
EPL	Pcp4, S100a5, Nrxn3, Doc2g, Camk2b, Fabp7, Apod, Cck, Atp1a2, Ppp3ca, Meis2, Nrsn1
MCL	Doc2g, Cck, Rab3b, Pcp4, Olfm1, S100a5, Nrsn1, Map1b, Cdhr1, Slc17a7, Fabp7, Nrxn3
ONL	S100a5, Fabp7, Apod, Pcp4, Ptgds, Ptn, Gng13, Gad1, Nrsn1, Omp, Doc2g, Plp1
RMS	Pcp4, Mbp, Nrxn3, Doc2g, Nrgn, S100a5, Tubb2b, Camk2b, Meis2, Nrsn1, Nrep, Mobp
GL	S100a5, Nrsn1, Pcp4, Fabp7, Apod, Calb2, Gng13, Ptgds, Cck, Nrxn3, Doc2g, Vsnl1
IPL	Pcp4, Atp1b1, Nrxn3, Doc2g, Nrsn1, Camk2b, Ppp3ca, Gad1, Scg2, Cck, Rab3b, Fxyd6
AOBgr	Nrxn3, Cpne6, Pcp4, Tac1, Doc2g, Fxyd6, Calb2, Plcb1, Camk2b, Dpp6, Jph4, Atp2b4
AOB	Fxyd6, Slc17a7, Rab3b, Pcp4, Stmn2, Uchl1, Doc2g, Cdhr1, Calb2, Cck, Nrxn3, Ptprd

* Here we assign clusters to the corresponding tissue structure: granule cell layer (GCL), external plexiform layer (EPL), mitral cell layer (MCL), olfactory nerve layer (ONL), rostral migratory stream (RMS), glomerular layer (GL), internal plexiform layer (IPL), granular layer of the accessory olfactory bulb (AOBgr) and accessory olfactory bulb (AOB) according to the ground truth.

Table S13. Marker gene selection of MuST on slice 151673 of DLPFC dataset acquired with 10x Visium.

Clusters	Marker genes
White Matter	MBP, PLP1, GFAP, CNP, PTGDS, TF, SNAP25, CRYAB, TMSB10, MOBP
Layer 1	MBP, GFAP, SNAP25, PLP1, MALAT1, TUBA1B, NEFL, CAMK2N1, STMN2, SLC24A2
Layer 2	MBP, CAMK2N1, TUBA1B, HPCAL1, NEFL, MALAT1, PLP1, TMSB10, ENC1, SOWAHA
Layer 3	MBP, PLP1, TMSB10, DIRAS2, GFAP, CAMK2N1, TUBA1B, NEFL, SNAP25, MALAT1
Layer 4	MBP, PLP1, TUBA1B, IGKC, IGLC2, GFAP, SNAP25, TMSB10, MALAT1, SCGB1D2
Layer 5	MBP, TMSB10, PCP4, PLP1, SNAP25, GFAP, TUBA1B, DIRAS2, HPCAL1, MALAT1
Layer 6	MBP, PLP1, DIRAS2, GFAP, PCP4, TMSB10, KRT17, SNCG, B3GALT2, SNAP25

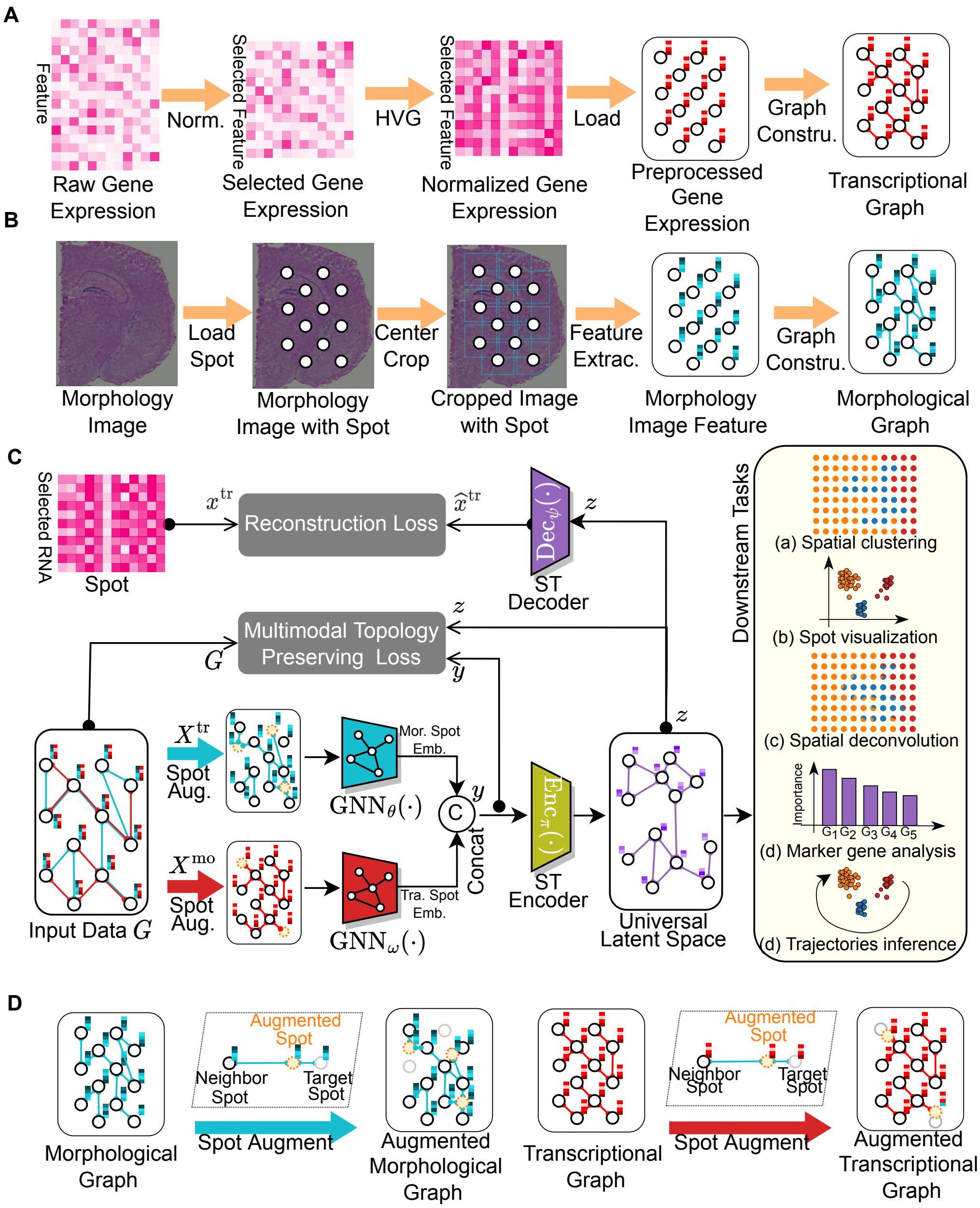

Fig. S1. Details framework of MuST. **A.** Preprocessing for transcriptomics modality. The gene expression counts are log transformed and normalised and then we select the 3000 most variable genes. **B.** Preprocessing for morphology modality. We centre and crop a 224 × 224 pixel area around the probe coordinates. These images are then processed by the ResNet50 architecture [56], to distill a 2048-dimensional feature vector and use PCA map the 2048-dimensional to 50-dimensional by PCA. **C.** Neural network data processing flow. **D.** Data augmentation of morphology and transcriptional.

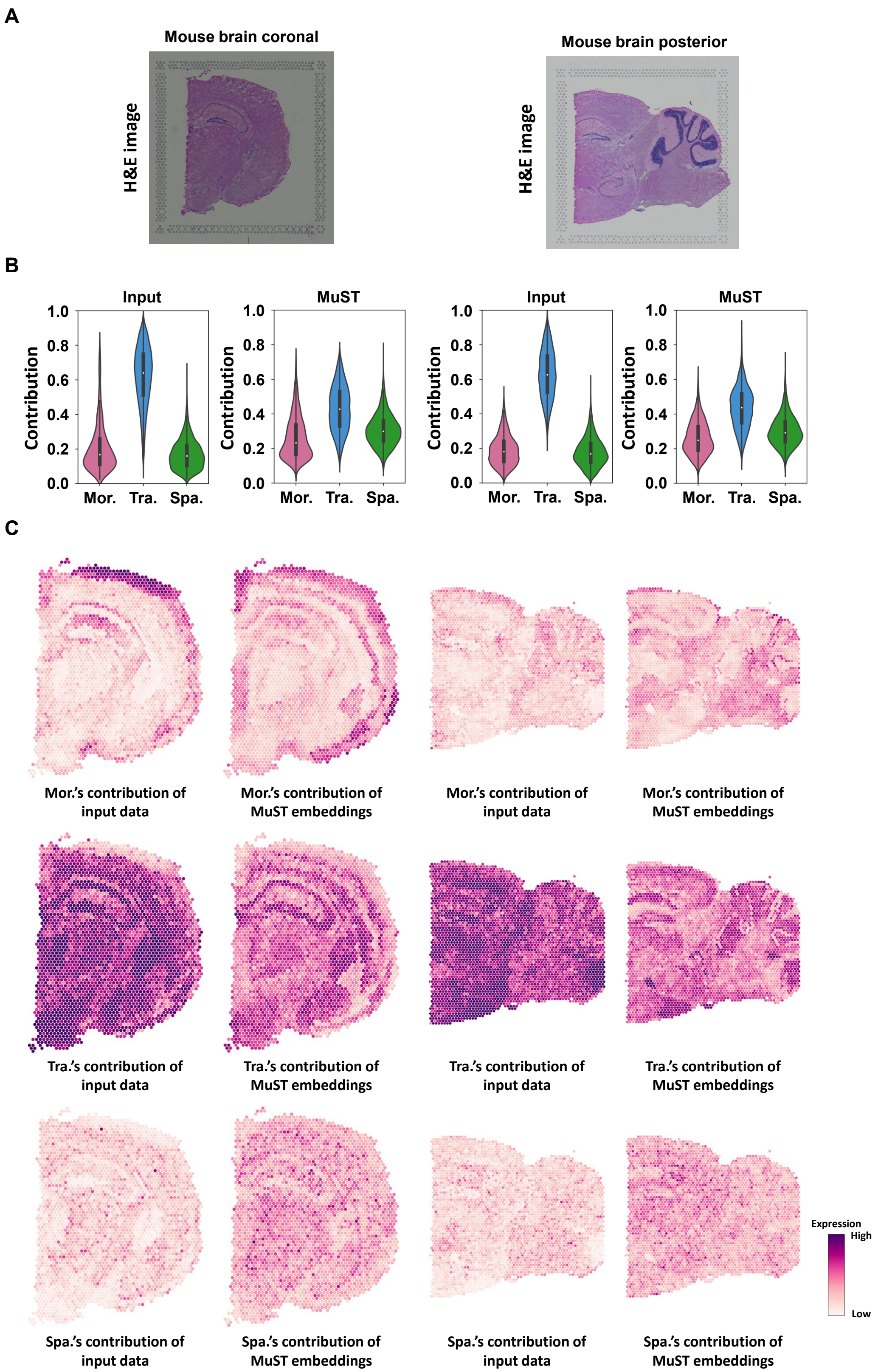

Fig. S2. MuST alleviates the modality bias phenomenon in mouse brain data. A H&E image of mouse brain coronal section and mouse sagittal posterior brain section. **B** Contribution of the morphology (Mor.) modality, transcriptome (Tra.) modality and spatial (Spa.) modality input data or MuST embedding to data labeling. **C** Contribution visualization of the Mor. modality, Tra. modality and Spa. modality input data or MuST embedding to data labeling.

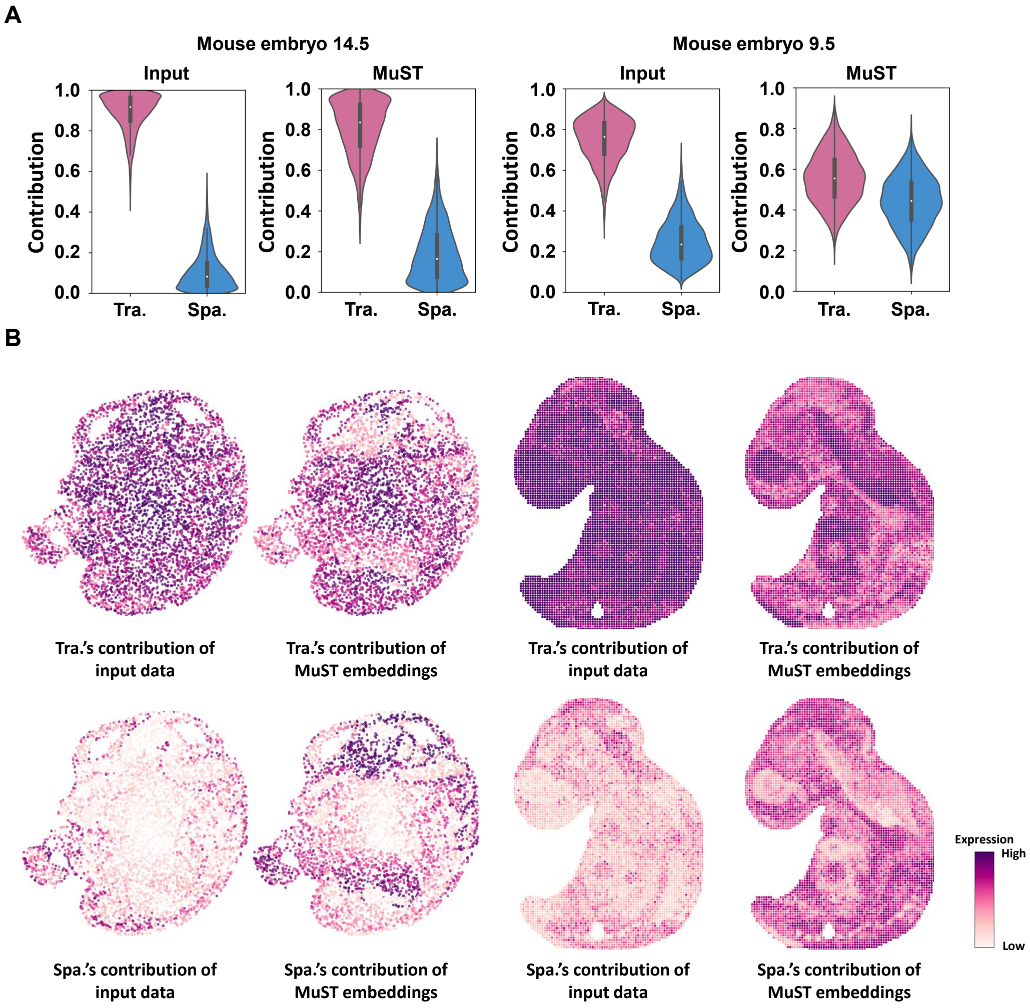

Fig. S3. MuST alleviates the modality bias phenomenon in mouse embryo data. **A** Contribution of the morphology (Mor.) modality, transcriptome (Tra.) modality and spatial (Spa.) modality input data or MuST embedding to data labeling on mouse embryo 14.5 and 9.5 datasets. **C** Contribution visualization of the Mor. modality, Tra. modality and Spa. modality input data or MuST embedding to data labeling.

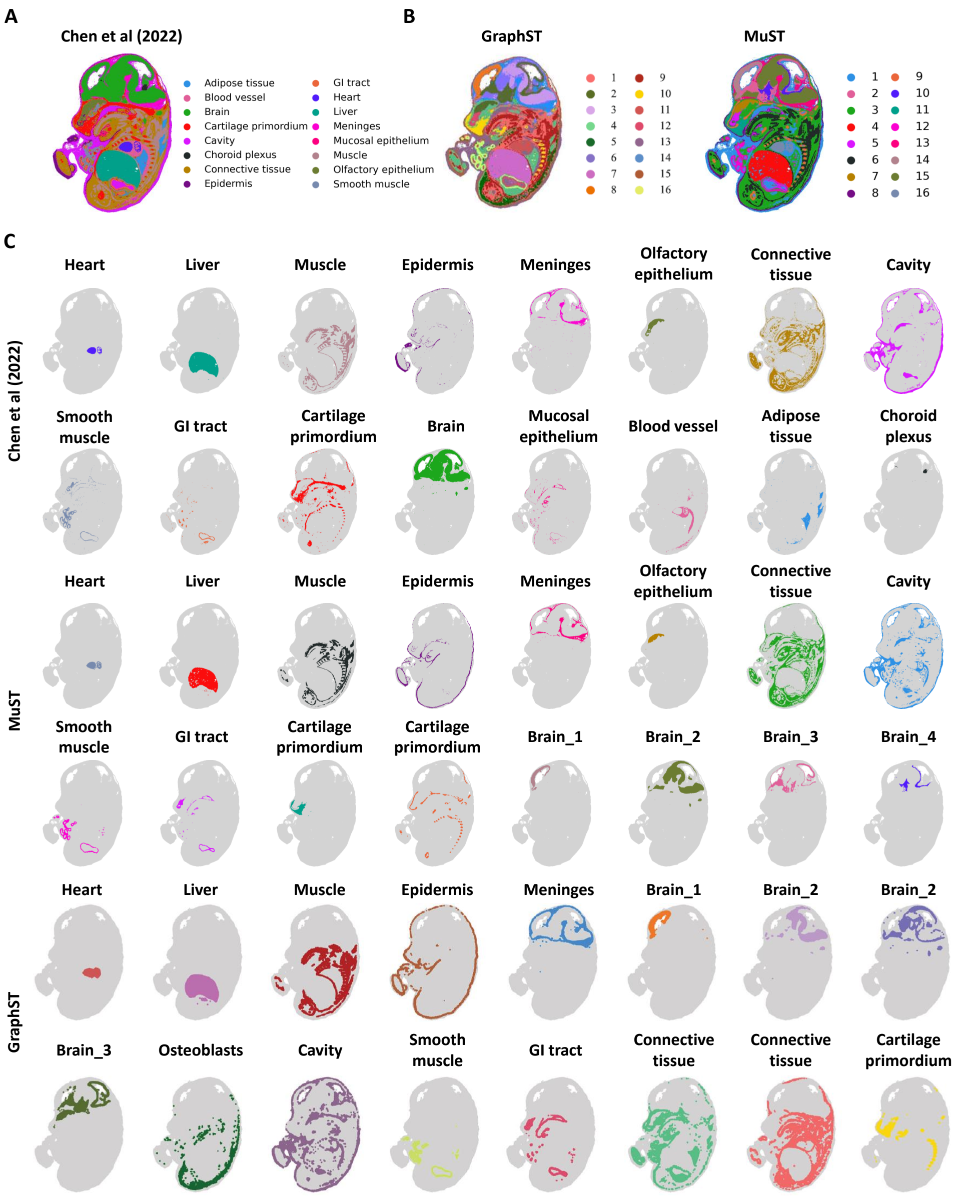

Fig. S4. MuST enables accurate identification of different organs in the Stereo-seq mouse embryo. A Tissue domain annotations of the E14.5 mouse embryo data obtained from the original Stereo-seq study. **B** Clustering results by GraphST and MuST on the E14.5 mouse embryo. **C** Cluster visualization of selected spatial domains identified by the original Stereo-seq study, MuST and GraphST, respectively.

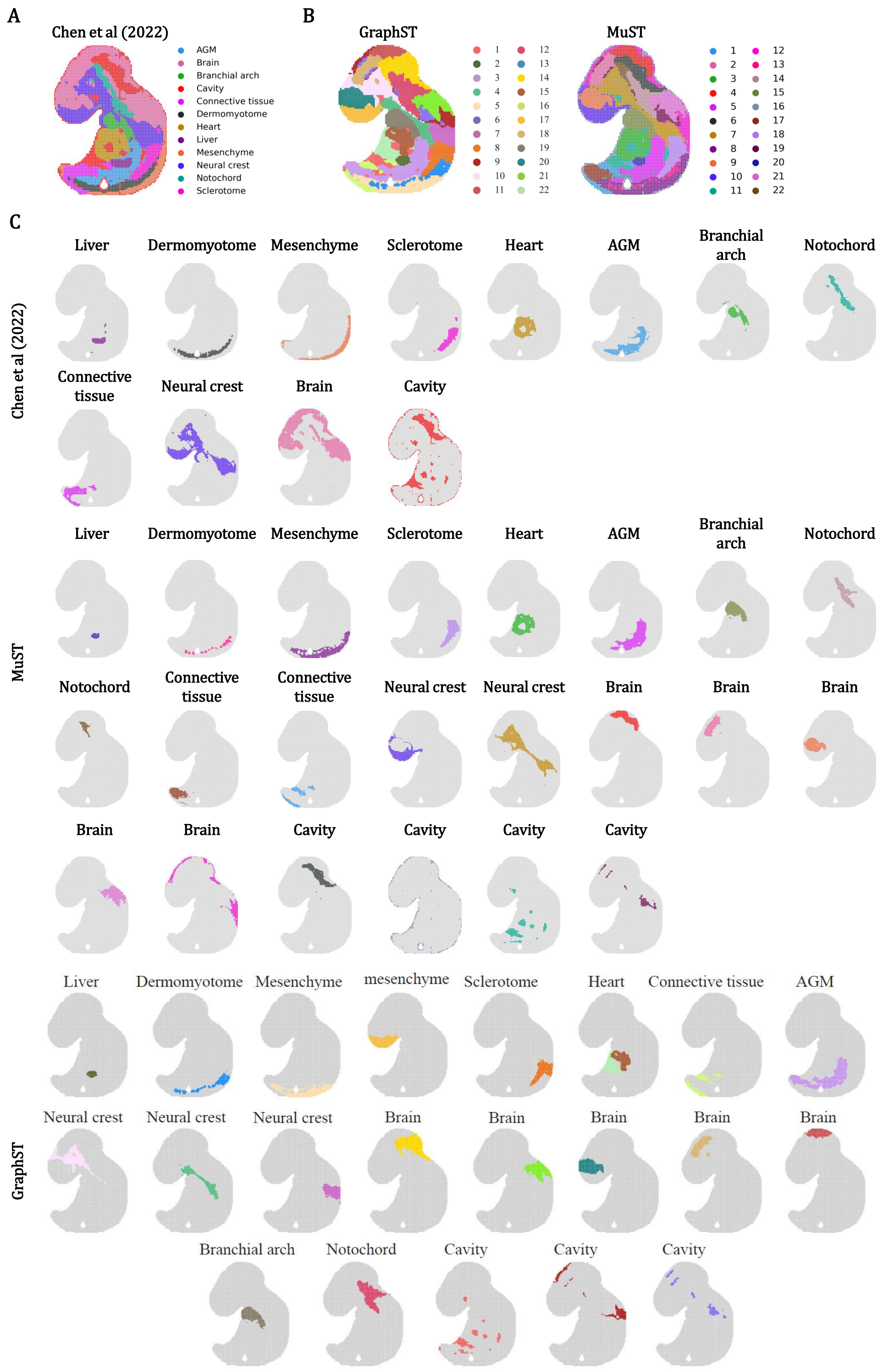

Fig. S5. MuST enables accurate identification of different organs in the Stereo-seq mouse embryo. **A** Tissue domain annotations of the E9.5 mouse embryo data obtained from the original Stereo-seq study. **B** Clustering results by GraphST and MuST on the E9.5 mouse embryo. **C** Cluster visualization of selected spatial domains identified by the original Stereo-seq study, MuST and GraphST, respectively.

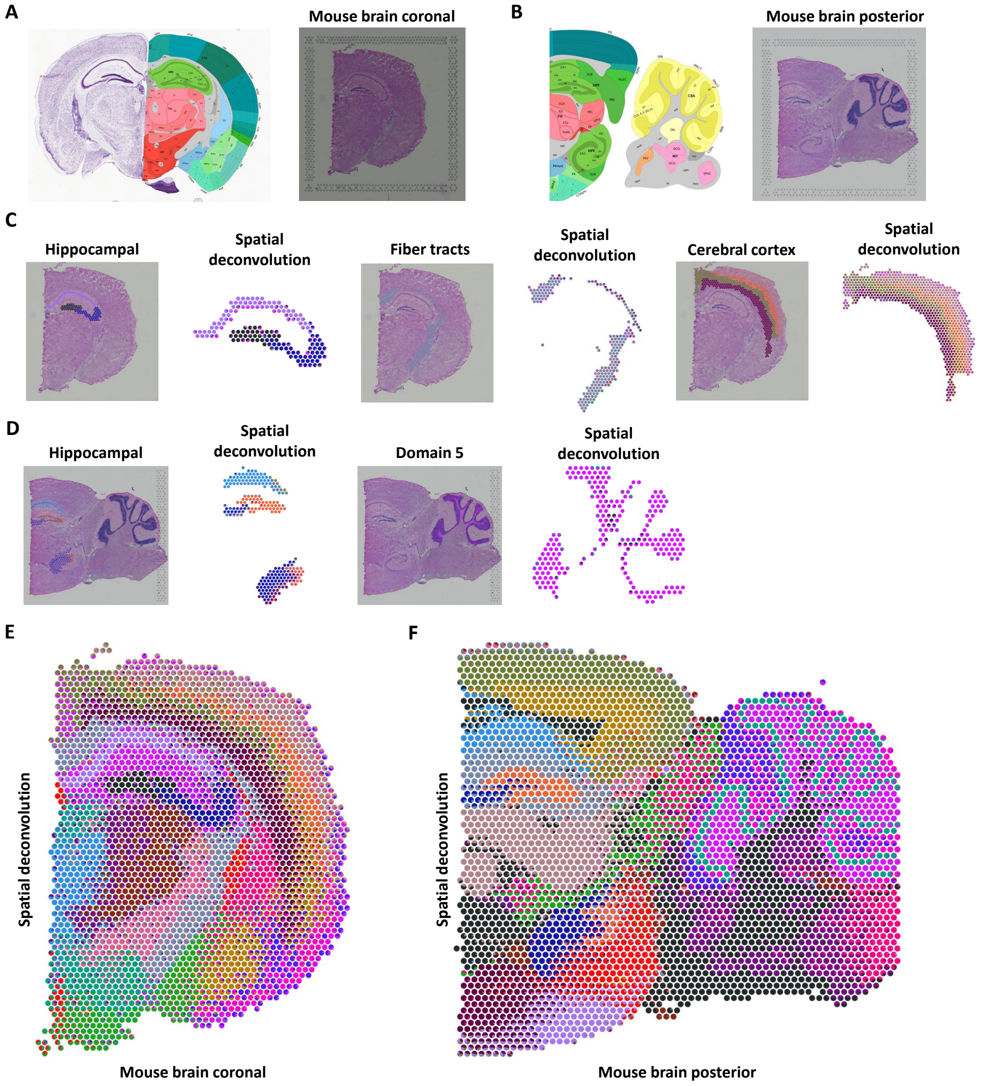

Fig. S6. Spatial deconvolution of MuST's results in adult mouse brain section profiled by 10x Visium. **A** Allen Brain Institute reference atlas diagram and H&E image of the mouse cortex. **B** Allen Brain Institute reference atlas diagram and H&E image of the mouse sagittal. **C** Spatial deconvolution of the identified domains by MuST on coronal mouse brain section. **D** Spatial deconvolution of the identified domains by MuST on mouse sagittal posterior brain section. **E** Spatial deconvolution of the coronal mouse brain section. **F** Spatial deconvolution of the mouse sagittal posterior brain section.

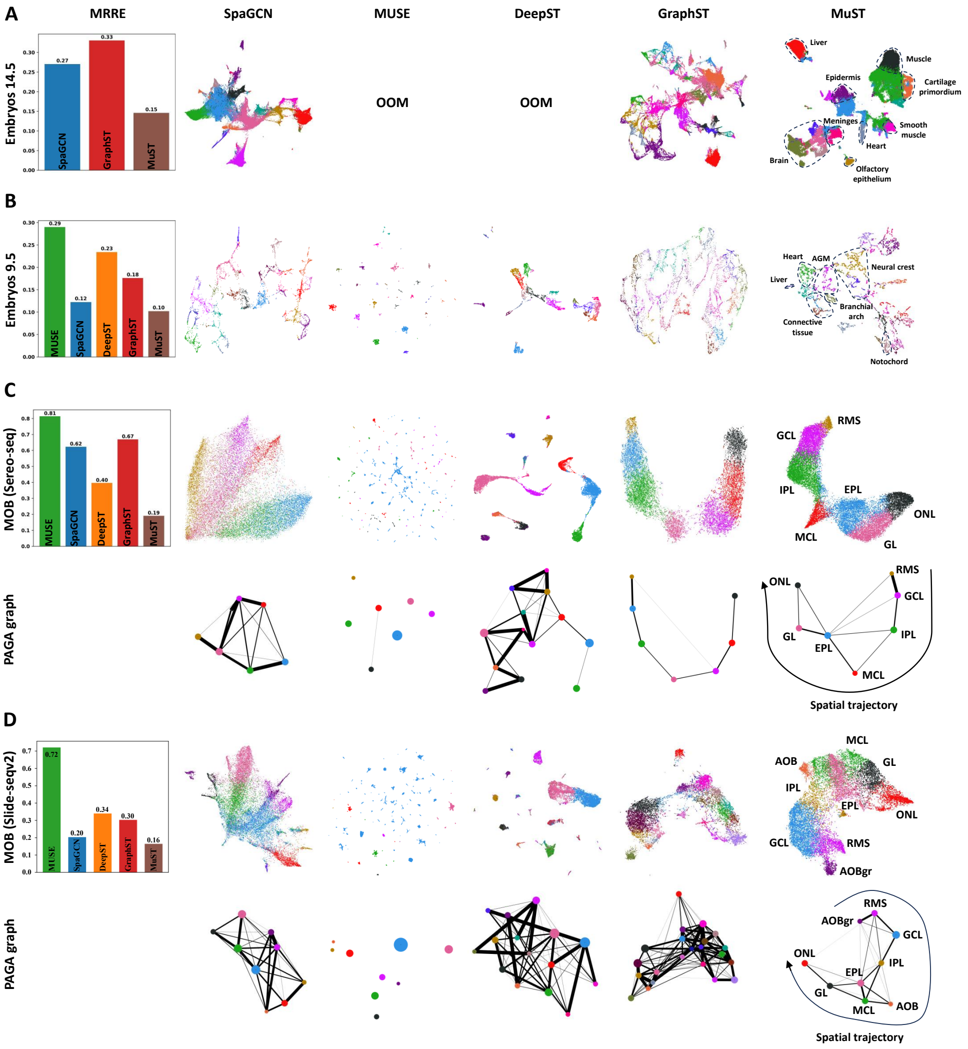

Fig. S7. Embedding analysis of MuST on the mouse olfactory bulb tissue and mouse embryo. A Mean relative rank error (MRRE) scores and UMAP visualizations generated by SpcGCN, MUSE, DeepST, GraphST and MuST representations on the Stereo-seq E14.5 mouse embryo data. Among them, methods MUSE and DeepST have an out of memory (OOM) problem. **B** MRRE scores and spot visualizations generated by the representations of five methods on the Stereo-seq E9.5 mouse embryo data. **C** MRRE scores, spot visualizations and PAGA graphs generated by the representations of five methods on the Stereo-seq mouse olfactory bulb tissue section. **D** MRRE scores, spot visualizations and PAGA graphs generated by the representations of five methods on the Slide-seqv2 mouse olfactory bulb tissue section.

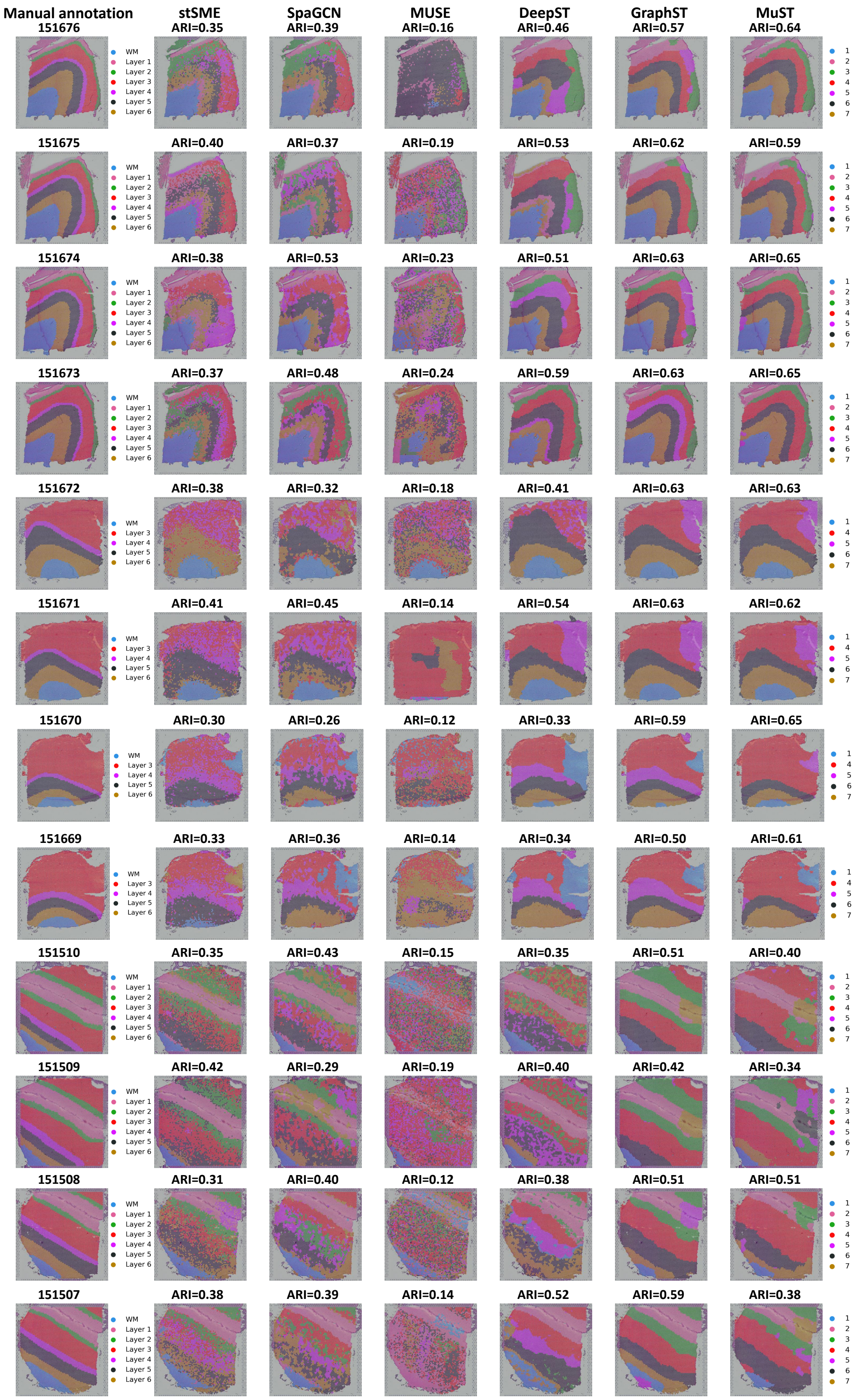

Fig. S8. Manual annotations and comparison of spatial domains identified by stSME, SpaGCN, MUSE, DeepST, GraphST and MuST on the 12 slices of DLPFC dataset.

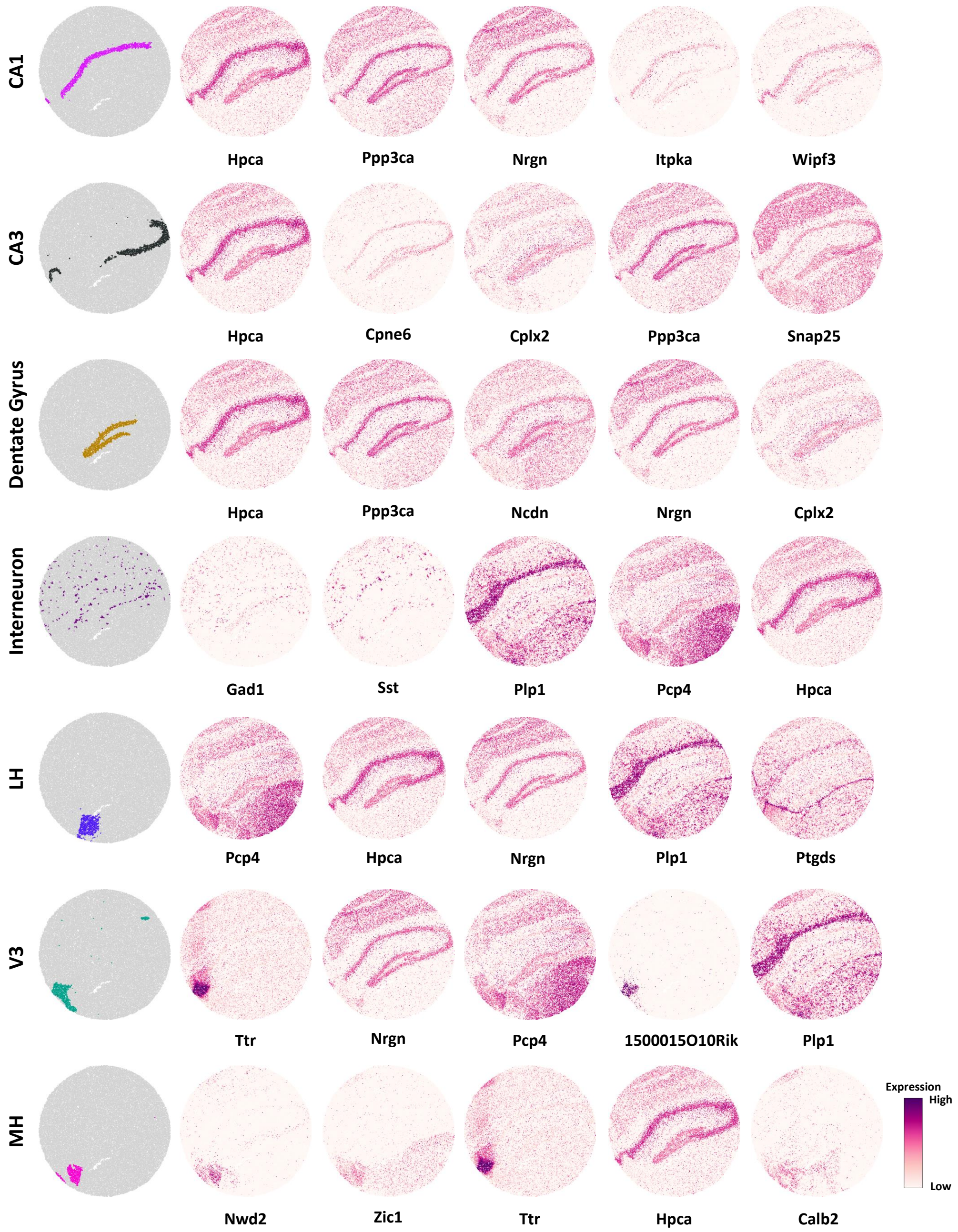

Fig. S9. Marker genes selection for each anatomical region of mouse hippocampus data acquired with SlideseqV2.

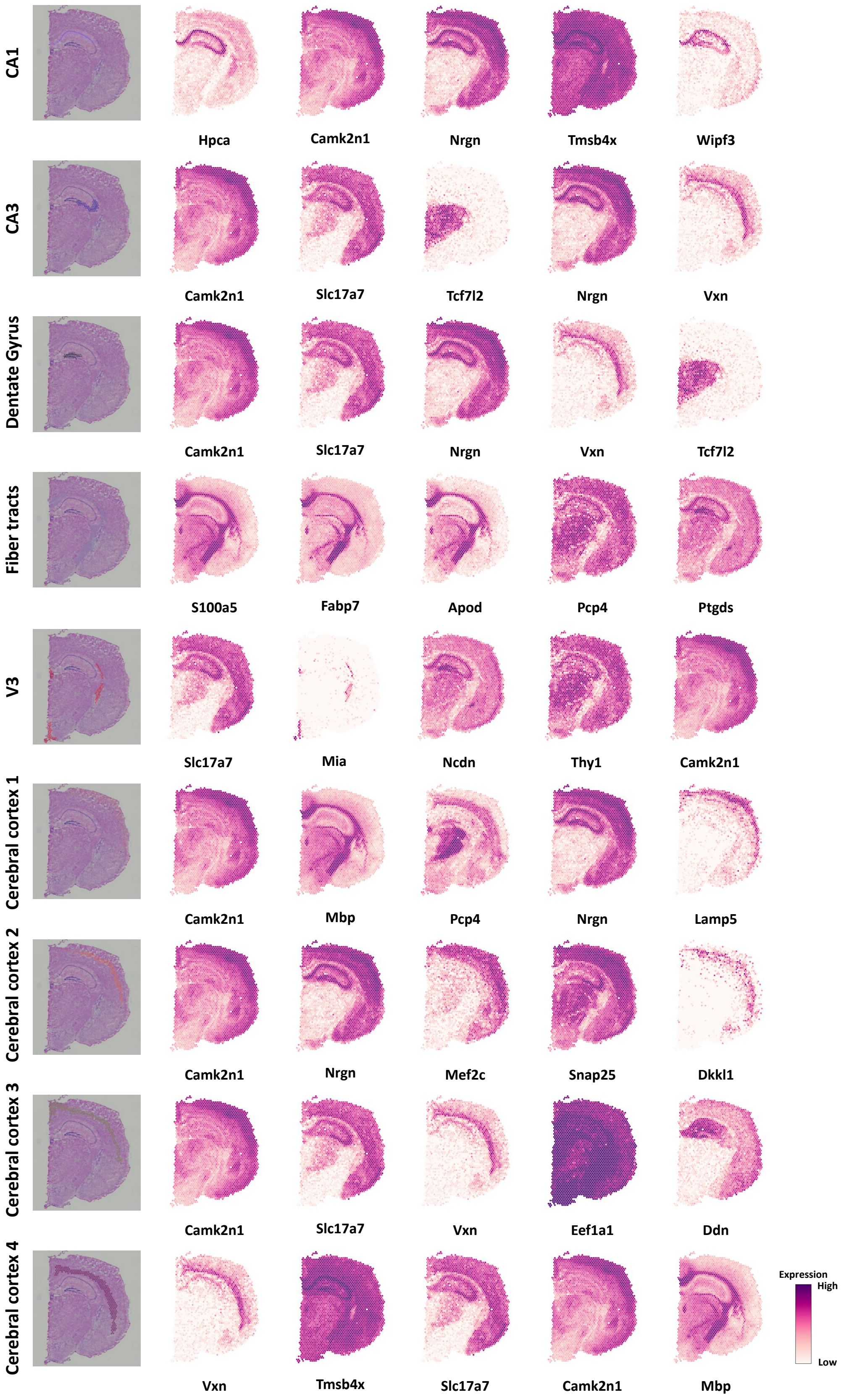

Fig. S10. Marker genes selection for each region of coronal mouse brain section acquired with 10x Visium.

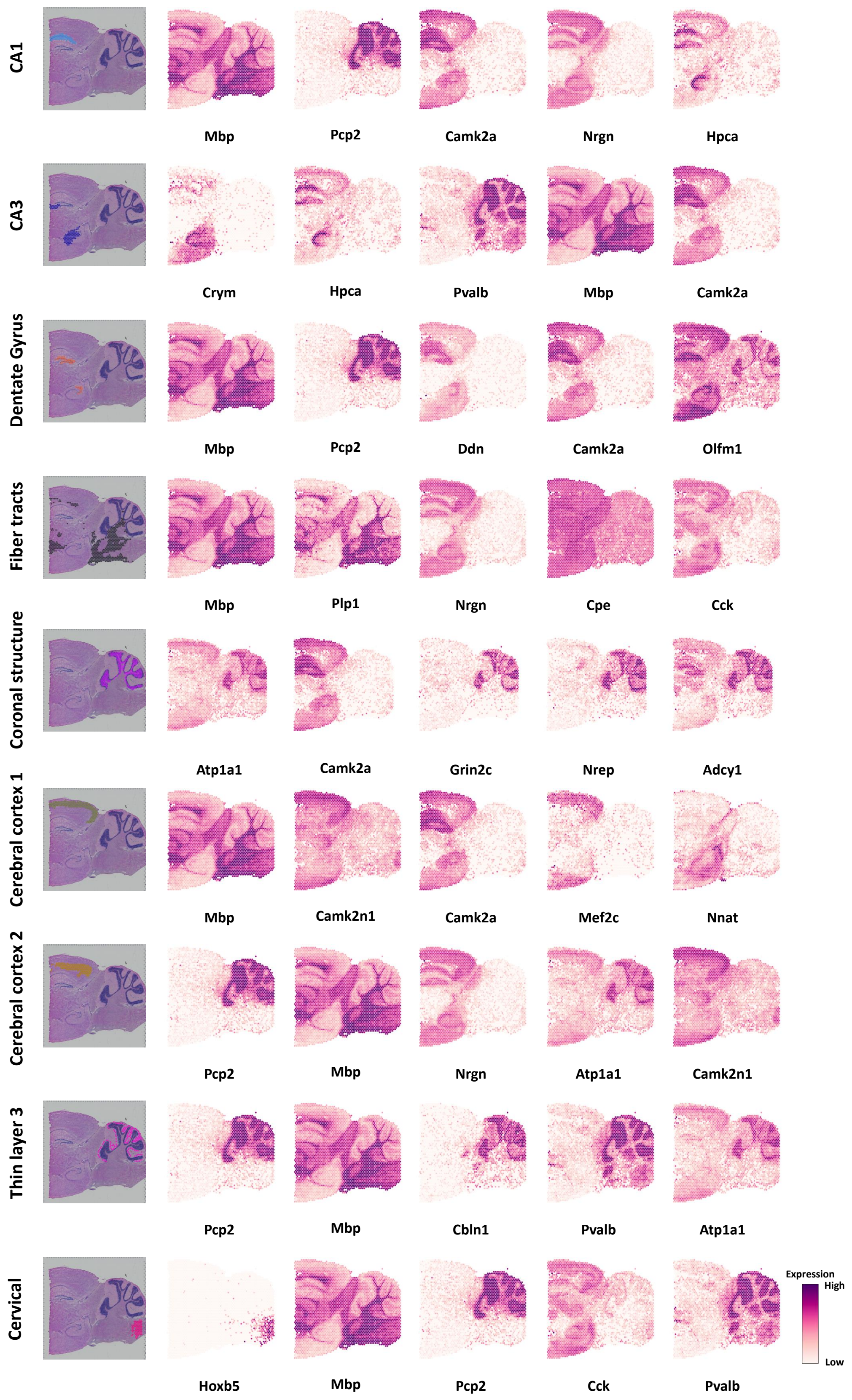

Fig. S11. Marker genes selection for each region of mouse sagittal posterior brain section acquired with 10x Visium.

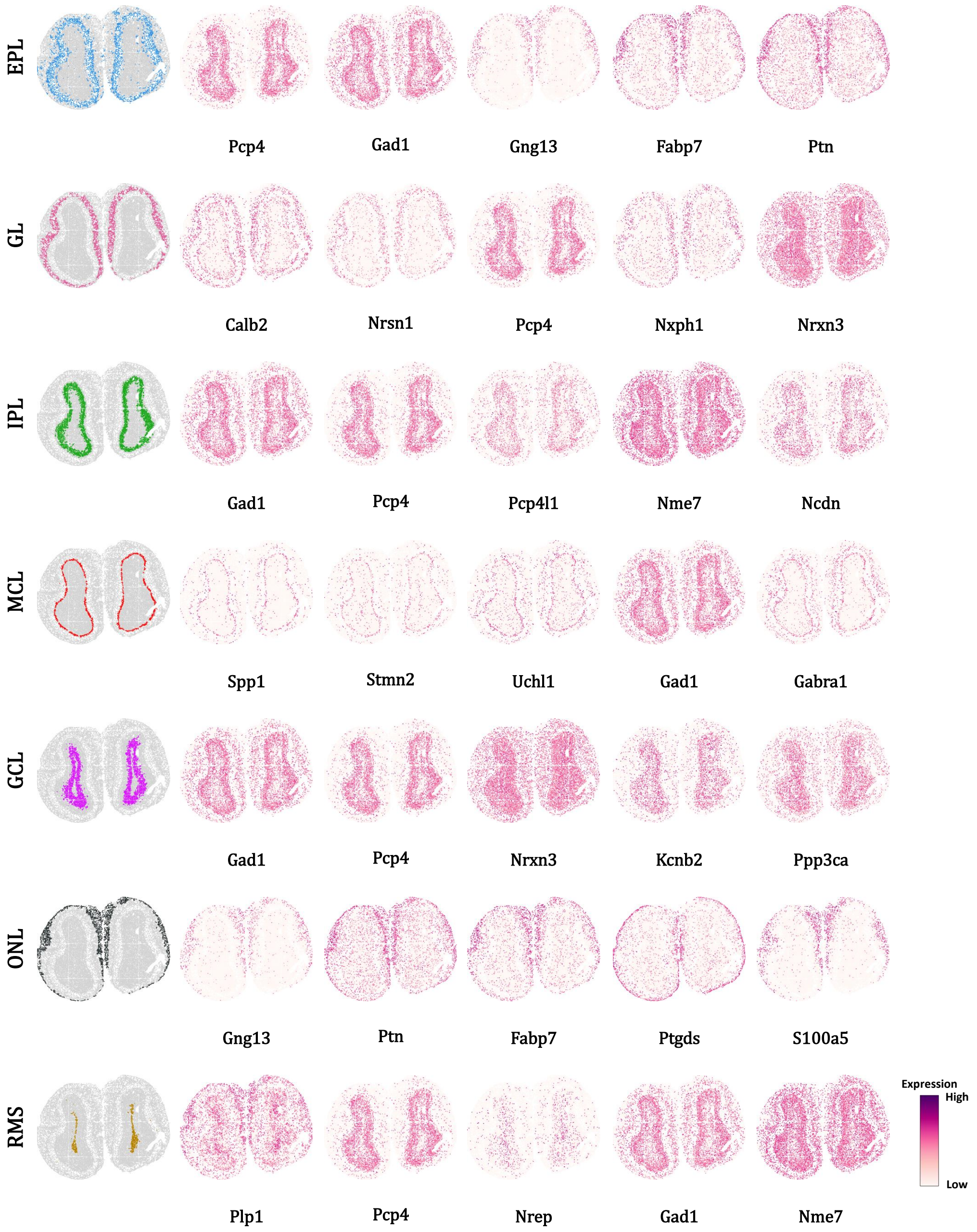

Fig. S12. Marker genes selection for each laminar organization of coronal mouse olfactory bulb tissue datasets acquired with Stereo-seq.

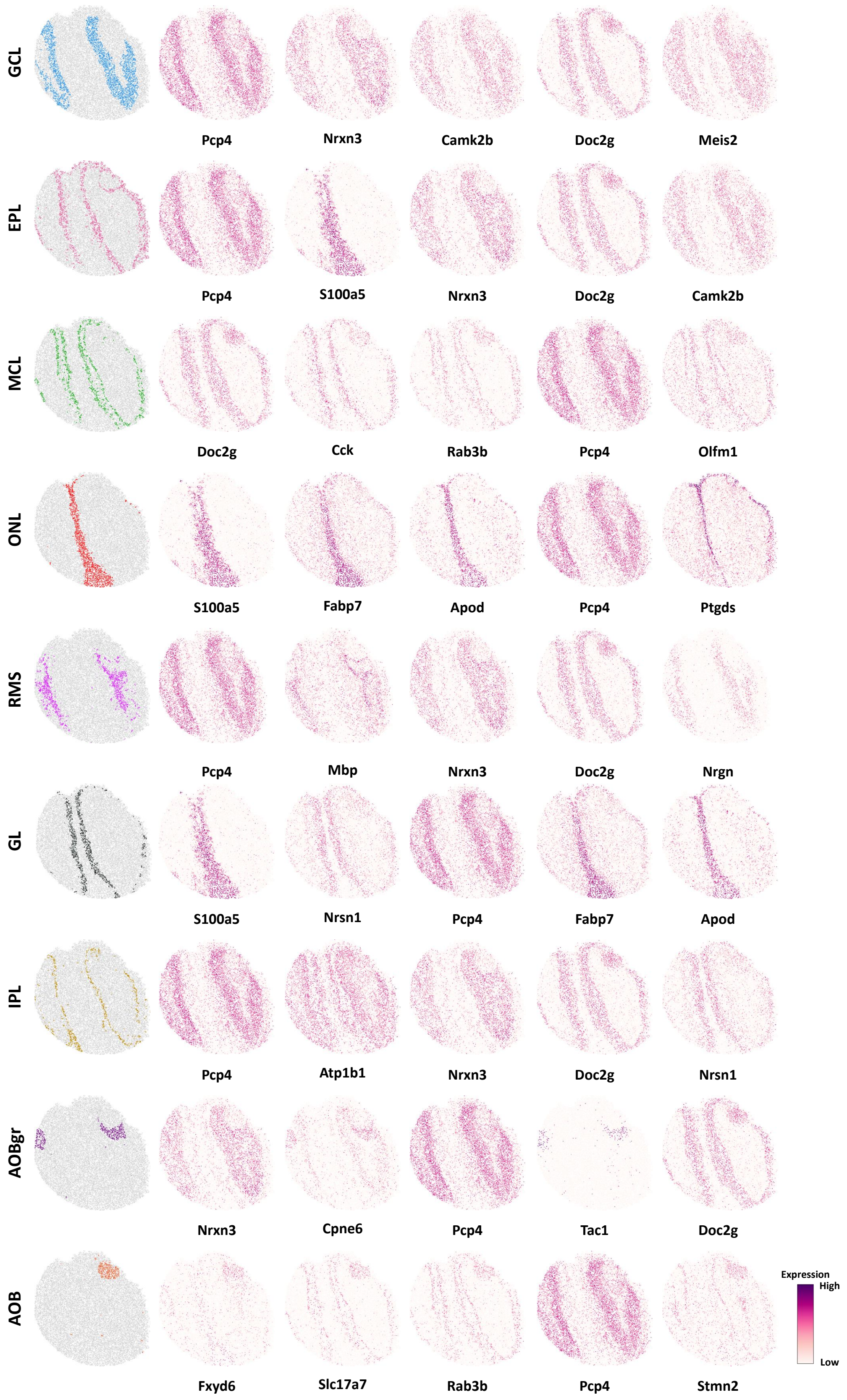

Fig. S13. Marker genes selection for each laminar organization of coronal mouse olfactory bulb tissue datasets acquired with Slide-seqV2.

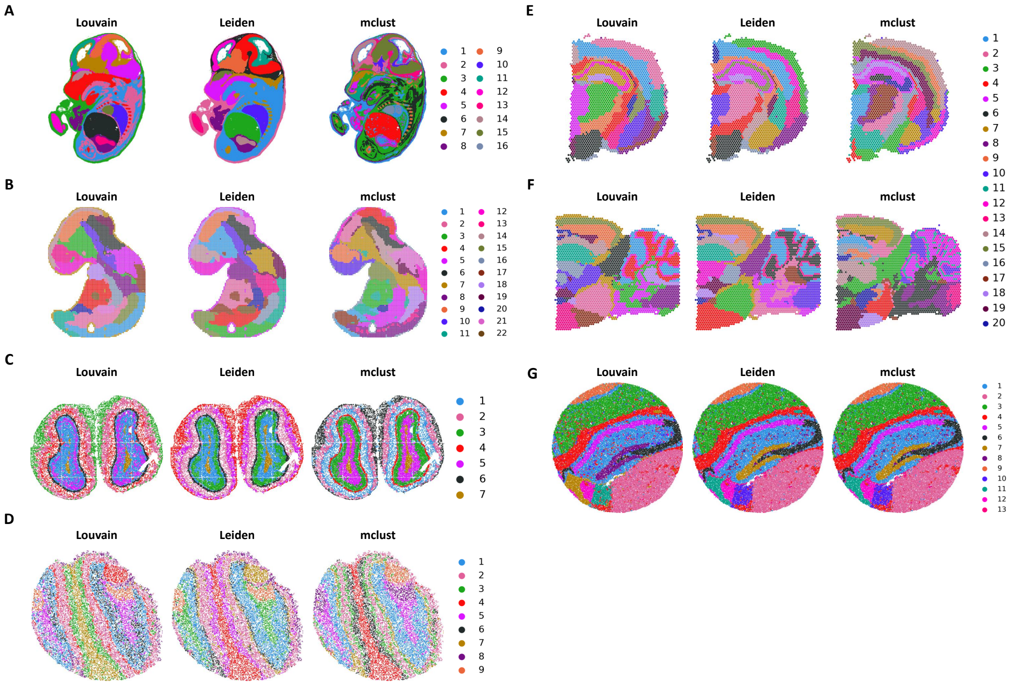

Fig. S14. Comparison analysis between Leiden, Louvain, and mclust with the output of MuST as input. Visualization of clustering results from Louvain, Leiden, and mclust on Stereo-seq E14.5 mouse embryo data (A), Stereo-seq 9.5 mouse embryo data (B), Stereo-seq mouse olfactory bulb tissue sections (C), Slide-seqV2 mouse olfactory bulb tissue sections (D), 10x Visium coronal mouse brain section (E), 10x Visium mouse sagittal posterior brain section (F), and SlideseqV2 mouse hippocampus data (G).
